# Chirality and asymmetry increase the potency of candidate ADRM1/RPN13 inhibitors

**DOI:** 10.1371/journal.pone.0256937

**Published:** 2021-09-10

**Authors:** Ravi K. Anchoori, Logan George, Ssu-Hsueh Tseng, Brandon Lam, Srinidhi Polkampally, Anjali D. Amiano, Palmer Foran, Hannah Tsingine, Harideep Samanapally, Fernanda Carrizo Velasquez, Samarjit Das, Deyin Xing, Ahmad Bin Salam, Balasubramanyam Karanam, Chien-Fu Hung, Richard B. S. Roden

**Affiliations:** 1 Department of Pathology, The Johns Hopkins University, Baltimore, Maryland, United States of America; 2 Department of Oncology, The Johns Hopkins University, Baltimore, Maryland, United States of America; 3 Department of Anesthesiology and Critical Care Medicine, The Johns Hopkins University, Baltimore, Maryland, United States of America; 4 Department of Biology and Center for Cancer Research, Tuskegee University, Tuskegee, Alabama, United States of America; 5 Department of Gynecology & Obstetrics, The Johns Hopkins University, Baltimore, Maryland, United States of America; B. S. Abdur Rahman Crescent Institute of Science and Technology, INDIA

## Abstract

Bortezomib and the other licensed 20S proteasome inhibitors show robust activity against liquid tumors like multiple myeloma, but have disappointed against solid tumors including ovarian cancer. Consequently, interest is mounting in alternative non-peptide based drugs targeting the proteasome’s 19S regulatory particle subunit, including its ubiquitin receptor RPN13. RA183 and RA375 are more potent analogs of the prototypic inhibitor of RPN13 (iRPN13) called RA190, and they show promise for the treatment of ovarian cancer. Here we demonstrate that rendering these candidate RPN13 inhibitors chiral and asymmetric through the addition of a single methyl to the core piperidone moiety increases their potency against cancer cell lines, with the S-isomer being more active than the R-isomer. The enhanced cancer cell cytotoxicities of these compounds are associated with improved binding to RPN13 in cell lysates, ATP depletion by inhibition of glycolysis and mitochondrial electron chain transport, mitochondrial depolarization and perinuclear clustering, oxidative stress and glutathione depletion, and rapid accumulation of high molecular weight polyubiquitinated proteins with a consequent unresolved ubiquitin proteasome system (UPS) stress response. Cytotoxicity was associated with an early biomarker of apoptosis, increased surface annexin V binding. As for cisplatin, BRCA2 and ATM deficiency conferred increased sensitivity to these iRPN13s. Ubiquitination plays an important role in coordinating DNA damage repair and the iRPN13s may compromise this process by depletion of monomeric ubiquitin following its sequestration in high molecular weight polyubiquitinated protein aggregates. Indeed, a synergistic cytotoxic response was evident upon treatment of several ovarian cancer cell lines with either cisplatin or doxorubicin and our new candidate iRPN13s, suggesting that such a combination approach warrants further exploration for the treatment of ovarian cancer.

## Introduction

The efficacy of the three licensed 20S catalytic particle (20S CP)-targeted covalent inhibitors (bortezomib, carfilzomib and ixazomib) against multiple myeloma and mantle cell lymphoma validate the proteasome as a cancer drug target. However, these proteasome inhibitors have not proven effective against solid tumors despite promising in vitro activity against representative cell lines. This implies that drug access may be the limiting factor, possibly due to their peptide backbones. Furthermore, they are associated with dose limiting side effects including peripheral neuropathy, thrombocytopenia, and neutropenia, as well as the eventual emergence of resistance. These limitations have driven drug discovery efforts to target different components of the proteasome with non-peptide inhibitors for the treatment of solid tumors.

The 19S regulatory particle (19S RP) inhibitors b-AP15 [1] and the more potent analog VLX1570 [2] are Michael acceptors that covalently bind to active site cysteine residues of two 19S RP proteins, USP14 and UCH37, and potentially other cellular targets [3]. Both possess significant antitumor properties in vitro and in animal models of solid cancers. Extended selection of cells in b-AP15 produced only very limited drug resistance (~2-fold) which was reversed by buthionine sulphoximine. This implies altered glutathione (GSH) metabolism as a resistance mechanism, whereas drug uptake and overexpression of drug efflux transporters did not contribute to the b-AP15 resistance acquired *in vitro* [4]. Mice lacking USP14 are viable but exhibit neuromuscular disease [5], whereas UCH37 deficiency causes embryonic lethality [6] consistent with b-AP15 and VLX1570 cytotoxic activity but raising questions about the breadth of the therapeutic window [7]. In a phase I in patients with relapsed and/or refractory multiple myeloma VLX1570 produced anti-myeloma effects at doses ≥0.6 mg/kg, but it lacked a sufficient therapeutic index [7].

Overexpression of *ADRM1* is an early event in the genesis of ovarian cancer [8], and correlates significantly with shorter time to recurrence and overall survival, as well as higher stage disease and recurrence [9]. *ADRM1* encodes RPN13, a ubiquitin receptor within the 19S regulatory particle (19S RP) of the proteasome. In one report, RPN13 knockout mice are viable [6], whereas in another study they died soon after birth [10], although a liver-specific knockout was tolerated [10] suggesting a useful therapeutic window may be achievable for an RPN13 inhibitor. The small molecule RA190 [11–13] was identified as the prototypic inhibitor of RPN13, and more potent analogs RA183 [14] and RA375 [15] were generated. Each of these molecules is a Michael acceptor that adducts RPN13 Cysteine 88 that resides within a groove required for binding of RPN13 to the proteasome via RPN2 [16, 17], as well as other cellular proteins at higher concentrations [18, 19]. However, in cytotoxicity assays, these RPN13 inhibitors (iRPN13) are not as potent as bortezomib based upon their IC50 against cancer cell lines. Glutathione (GSH) metabolism counteracts the cytotoxic effects of RA183 and RA190. The addition of chloroacetamide moiety to these compounds (RA375 and RA371 respectively) enhanced their potency by reacting with, and thereby further reducing intracellular GSH and its completion for binding to RPN13 [15], but the introduction of chloroacetamide also reduces drug specificity and aqueous solubility [15].

In seeking compounds with higher potency and specificity towards RPN13, here we rationally designed new analogs using molecular modeling and knowledge of RA183’s pharmacophore. In particular, we examined the impact of addition of a methyl group to the core piperidone moiety, which renders the molecule (RA413) chiral and asymmetric, upon mechanism of action and activity in vitro and in vivo.

## Materials and methods

### Cell lines and cytotoxicity assays

All cell lines were obtained from the American Type Culture Collection (ATCC) and cultured in the specified medium supplemented with 10% fetal bovine serum, 100 IU/mL penicillin, and 100 μg/mL streptomycin at 37°C in a humidified 5% CO_2_/95% air incubator. Synthesis of key compounds is described in S1 File, and >95% purity of RA413S and RA414 were confirmed by nuclear magnetic resonance (NMR) and mass spectroscopy (MS) analyses. To assess drug cytotoxicity, cells were seeded at 2,500 cells/well (10,000 cells/well for MM lines) in 100 μL medium in 96-well plate and after 24h treated with compounds for 72h, incubated according to the manufacturer’s protocol with the Thiazolyl Blue Tetrazolium Bromide (Sigma, M2128), and A_570_ measured using a Benchmark Plus microplate spectrophotometer (BIO-RAD). IC50 data was analyzed by Graphpad Prism software v.8.2.0.

### Antibodies, reagents and Western blot analyses

Cell lysate (10–20 μg total protein) prepared in MPER (Pierce) from each sample was subjected to SDS-PAGE, transferred to PVDF membranes and analyzed by Western blot using antibodies specific to ubiquitin (P4D1, sc-8017, Santa Cruz), actin (#66009, Protein Tech Group), Tubulin (#66031, Protein Tech Group), ADRM1/Rpn13 (D9Z1U, #12019, Cell Signaling) caspase-3 (51-68655X, BD Pharmingen), Annexin V (#559763, BD pharmingen) and for secondary antibodies we utilized either peroxidase-linked anti-mouse IgG or anti-rabbit IgG (GE Healthcare UK Ltd) at the recommended dilution (1:5000), MitoTracker Red CMXRos reagent was purchased from ThermoFisher #M7512.

### Reporter assays

Sub-confluent cultures of cells were transfected with 4Ub-FL or FL plasmid using TransIT 2020 reagent (Mirus Bio). Cells were seeded at 10,000 cells/well in 96-well plates 48 h post-transfection and incubated with compounds or vehicle (DMSO) at the doses and times indicated. Luciferase activity in cell lysate was determined with a luciferase assay kit (Promega) according to the manufacturer’s instructions. Bioluminescence was measured by using a Glomax Multidetection system (Promega).

### ATP measurement assay

To measure ATP levels in cells, a luciferase-based bioluminescence assay was performed. Briefly, cells stably expressing firefly luciferase were grown in a 6 well plate (250,000 cells/well) for 24 h and then treated with compounds at indicated concentrations for 4 h, whereupon the bioluminescence was measured in an IVIS 200 imager in intact cells after addition of luciferin (0.75 mg in 100 μL PBS) to the medium. Cell viability was assessed in parallel using an MTT assay.

### Flow cytometry analysis of cell death

10^5^ cells were re-suspended in binding buffer, 5 μL of Annexin V-PE (Apoptosis Detection Kit I (BD Pharmingen) and 5 μL of 7-AAD were then added for 15 min at room temperature before flow cytometric analysis on a FACSCalibur using CellQuest software (Becton Dickinson).

### Amplex Red assay to measure ROS

Briefly, cells were treated in 96 well plates with corresponding compounds for the indicated time periods. Next, 50 μM Amplex^®^ Red (#A12222, Thermo Scientific) and 1 U/mL horseradish peroxidase (HRP) in 50 mM sodium phosphate buffer, pH 7.4, were added to cells and incubated for 30 minutes at room temperature. Fluorescence was measured with a fluorescence-based microplate reader using excitation at 530 ± 12.5 nm and detection at 580 ± 25 nm. Background fluorescence of 969 units for Amplex^®^ Red reagent, determined for a no-H_2_O_2_ control reaction, was subtracted from each value. H_2_O_2_ treatment (1 h) was used as a positive control.

### Biotin labeling assay

Clarified SKOV3 cell lysate in MPER buffer (5X10^6^ cells/mL) was pretreated with 40 μL Dyna beads MyOne streptavidin T1 magnetic beads (Invitrogen 65602) for 45 min at 4°C to deplete non-specific biotinylated proteins in the cell lysate. After separation of the beads, 40 μL of the pre-cleared cell lysate was incubated with compounds (20 μM) for 45 min at 4°C, and then treated with RA183B or RA413SB (10 μM) for 45 min at 4°C. Next, the samples were mixed with Laemmli sample buffer (BioRad) and boiled for 5 min. The proteins were separated using a 4–15% Bio-Rad Mini-PROTEAN SDS-PAGE gel (1 h at 100 V), and transferred to PVDF membrane overnight at 4°C (24 V). The membrane was blocked with 5% BSA in PBST for 1 h at RT and washed for 20 minutes (3X with PBST). The membrane was probed with HRP-streptavidin (N100, Thermo Fisher, 1:10,000 in PBST) for 1 h at RT, washed for 30 min (3X with PBST), and developed using HyGLO chemiluminescent detection reagent (Denville) with imaging on a Biorad Gel Doc for biotin detection. For bacterial expression, *RPN13*-pET28a (+) was transformed into *E*. *coli* (DE3) Rosetta 2 cells (Novagen) and cultures in LB and kanamycin at A_600_ ~0.6 were induced with 0.4 mM IPTG for 4 h at 37°C. Cell pellets were treated with BPER lysis buffer 1X cOmplete^™^, EDTA-free Protease Inhibitor Cocktail, and 100 μg/mL lysozyme for 30 min on ice. The lysates were clarified by centrifugation and frozen. Equal amounts of cell lysate (determined by BCA, ~40 μg) were treated with DMSO or different concentrations biotinylated compounds (RA190B, RA183B and RA413SB) for 45 min at 4°C and subjected to SDS-PAGE, transfer to PVDF membrane and probed with HRP-streptavidin as described above.

### Q-PCR to measure mRNA levels

Total RNA was isolated from cells using an RNeasy mini kit (Qiagen). Extracted RNA was normalized for concentration and reverse transcribed using an iScript cDNA synthesis kit (Bio-Rad). *CHOP10* expression levels were measured by Taqman gene expression assays with Taqman gene expression master mix (Applied Biosystems) and run with a standard thermal cycling protocol. Calculations were done according to the Livak method and normalized to the reference gene GAPDH. Each condition was replicated three times; each sample was run in triplicate.

### MitoTracker assay

HeLa cells were seeded at 1 x 10^5^ /well into 12-well plates containing glass coverslips and grown overnight to reach 50–70% confluence. Cells were treated with RA413 (100 nM and 500 nM; 6, 8, and 12 hours), CCCP (1 μM; 30 minutes), bortezomib (500 nM; 6, 8, and 12 hours) suspended in DMSO, or DMSO as a vehicle control (6, 8, and 12 hours). Following the time course, media was replaced with fresh media containing 100nM MitoTracker Red CMXRos (from a stock in DMSO) and incubated for 45 minutes. Cells were fixed with -20°C 100% methanol for 15 minutes then washed three times with 1x PBS. DNA was stained with Hoechst (1:5000) in PBS for 10 minutes and washed three times with 1x PBS. Coverslips were mounted on glass microscope slides using ProLong Glass Antifade Mountant. Coverslips were imaged using a Nikon epifluorescent microscope using a Nikon 60x Plan Apo Oil objective. Imaging of each condition was conducted in at least three individual experiments.

### Mitochondrial functionality analysis

mtDNA quantification: Cells were grown in 100 mm dishes overnight to 90% confluence. Cells were treated with RA413 (100 nM, 250 nM, or 500 nM), bortezomib (500 nM), or DMSO vehicle for 18 hours. Following treatment, cells were trypsinized and centrifuged into microfuge tubes. Total DNA was extracted using the DNeasy Blood and Tissue Kit (Qiagen) by the manufacturer’s protocol. Samples were collected in triplicate. DNA purity and concentration was measured with Nanodrop. mtDNA content was measured by multiplexing real-time PCR using a QuantStudio 5 reader (Thermo) and the TaqMan Fast Advanced Master Mix (Thermo). The primers used for the mitochondrial gene was mtCOX1 in FAM dye (Assay ID: Mm04225243_g1) and the nuclear gene GAPDH in VIC dye (Assay ID: Ms999915_g1). Total DNA in the PCR mixture was 5 ng. Amplification conditions were 50°C for 2 min, 95°C for 2 min, and 40 cycles of 95°C for 1 s and 60°C for 20 s. The mtDNA/nDNA ratio of 2ΔdCt is reported as mitochondrial number.

### Seahorse assay

For quantification of oxygen consumption rate, ES2 cells were seeded at 20,000/well in Seahorse plates in RPMI-8226 medium and incubated at 37°C with 5% CO_2_ overnight. After 8 h of treatment with the compounds, the cells were washed with PBS and then returned to Seahorse culture media (RPMI + 17.5 mm glucose + 1 mM pyruvate + 2 mM glutamine). The plate was then incubated at 37°C without CO_2_. After 1 h incubation, mitochondrial respiration was measured using a Seahorse XF96 Analyzer. The oxygen consumption rate (OCR) and extracellular acidification rate (ECAR) were analyzed before and after adding oligomycin (3 μM), FCCP (4 μM), antimycin (2 μM), and rotenone (2 μM), sequentially. The OCR and ECAR data were normalized with the protein concentration, and further analyzed using Seahorse Wave 2.4 software.

### Schrödinger methods: Glide ligand docking

The ligands RA413S, RA183 and RA414 were docked into RPN13 Pru Domain protein (PDB ID: 5IRS) by utilizing the Glide module of Schrödinger suite Maestro V12.3. The docking was performed utilizing Standard precision (XP) mode and OPLS-3e power field and the above docking process was run in a flexible docking mode which automatically generates conformations for each input ligand. The best-docked ligands are chosen based on MM-GBSA score using Prime-MM-GBSA module of Schrodinger Maestro Suite 2020–2.

### Animal studies

All animal procedures were performed according to protocols pre-approved by the Johns Hopkins University Animal Care and Use Committee, and in accordance with the AAALAC recommendations for the proper use and care of laboratory animals (protocol MO18M129). Four to six week old female Nude (002019), or CD1 mice or C57BL6 mice were purchased from Jackson Laboratories (ME, USA), and acclimatized to the facility for ≥1 week. Isoflurane anesthesia was used during imaging. The color of the drug precluded blinding. The health conditions and/or criteria under which early euthanasia or withdrawal of an animal from the study was implemented included, but are not limited to, general signs of distress such as hunched posture, lethargy, anorexia, dehydration, rough hair coat and/or those that are directly related to the experimental procedures e.g. loss of weight >10%, lethargy, restricted movement of limbs, distended abdomen. Animals in distress were euthanized by carbon dioxide asphyxiation, and cervical dislocation was used to ensure death. This is an acceptable form of euthanasia for mice in compliance with the recommendations of the Panel on Euthanasia of the American Veterinary Medical Association.

### Electroporation method

A patch of CD1 mouse leg was shaved of hair and 10 μg 4Ub-FL plasmid in 20 μL of PBS was injected into the *quadriceps femoralis* muscle followed immediately by injection of the 2 Needle Array to 5 mm depth encompassing the injection site and square wave electroporation (ElectroSquarePorator 833, BTX-2 Needle array 5mm gap, Harvard apparatus) delivered as eight pulses at 106V for 20 ms with 200 ms intervals. One day post electroporation, mice were anesthetized with isoflurane, injected i.p. with luciferin (0.3 mg in 100 μL water) and optical imaging was performed to determine basal level luciferase expression. Images were acquired for 10 min with a Xenogen IVIS 200 (Caliper, Hopkinton, MA). Equally sized areas were analyzed using Living Image 2.20 software.

### Tumor studies

Female Nude or C57BL6 mice were inoculated respectively with 10^6^ ES2-Luciferase or ID8-Vegf *Defb29* syngeneic mouse model of ovarian cancer expressing luciferase [20] *i*.*p*. in 100 μL PBS. At day 3, mice were imaged for basal level luminescence activity with a Xenogen IVIS 200 after injection *i*.*p*. with luciferin (0.3 mg in 100 μL water). Mice were randomized into two groups and treated *i*.*p*. with compounds or vehicle (25% (*w*/*v*) β-Hydroxypropylcyclodextrin in water), and imaged again on days 7 and 14.

### Statistical analyses

Results are reported as mean ± standard deviation (s.d.). Statistical significance of differences was assessed by two-tailed Student’s *t* using Prism (V.8.2.0 Graphpad, San Diego, CA) with the level of significance set at p≤0.05. Survival was summarized using Kaplan–Meier methods and compared using log-rank tests. Combination indices (CI) were calculated using SynergyFinder 2.0: visual analytics of multi-drug combination synergies.

## Results and discussion

### Optimization strategy to design novel RPN13 inhibitors

**Fig 1:**
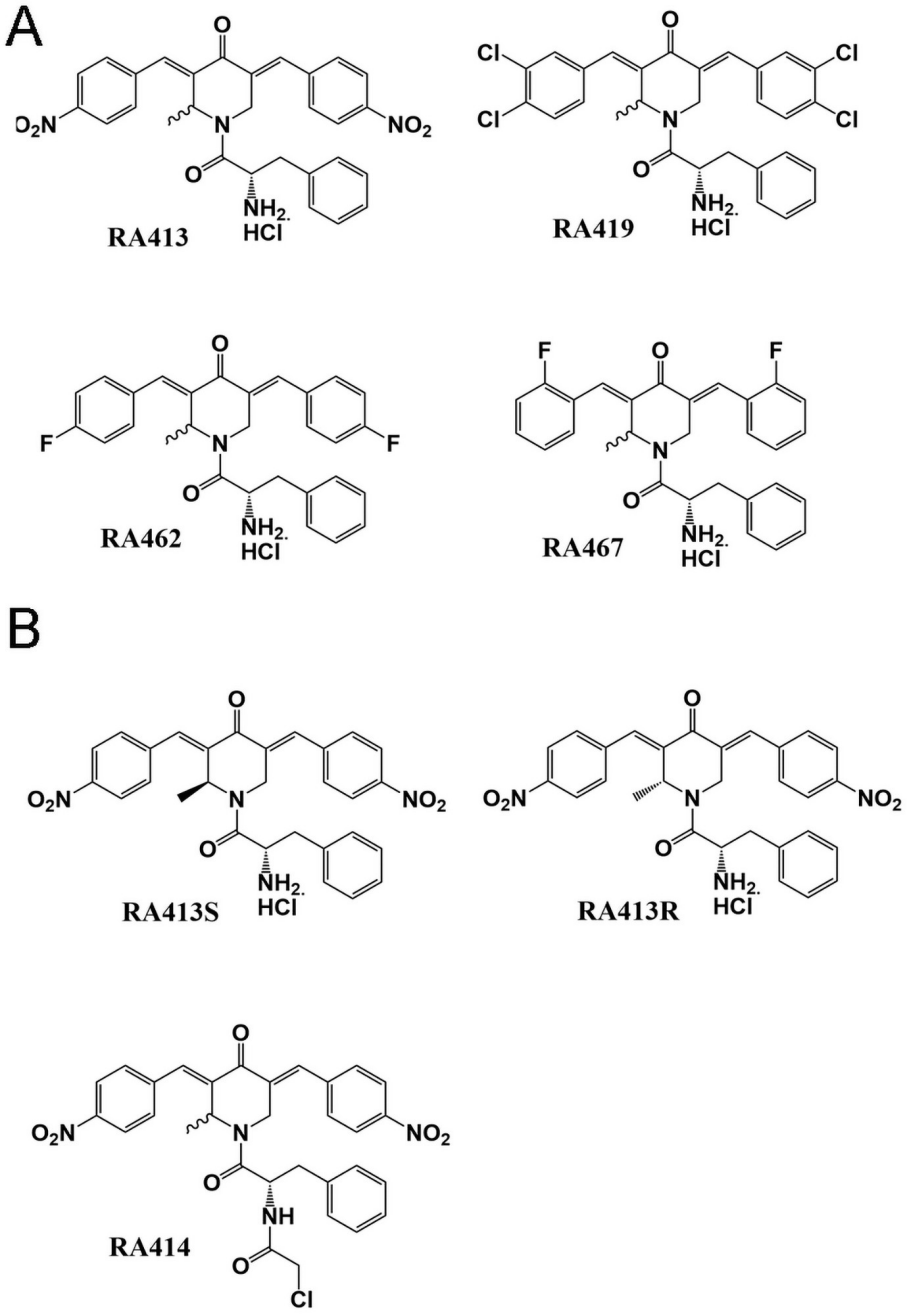
Chemical structures of inhibitors.

**Fig 2:**
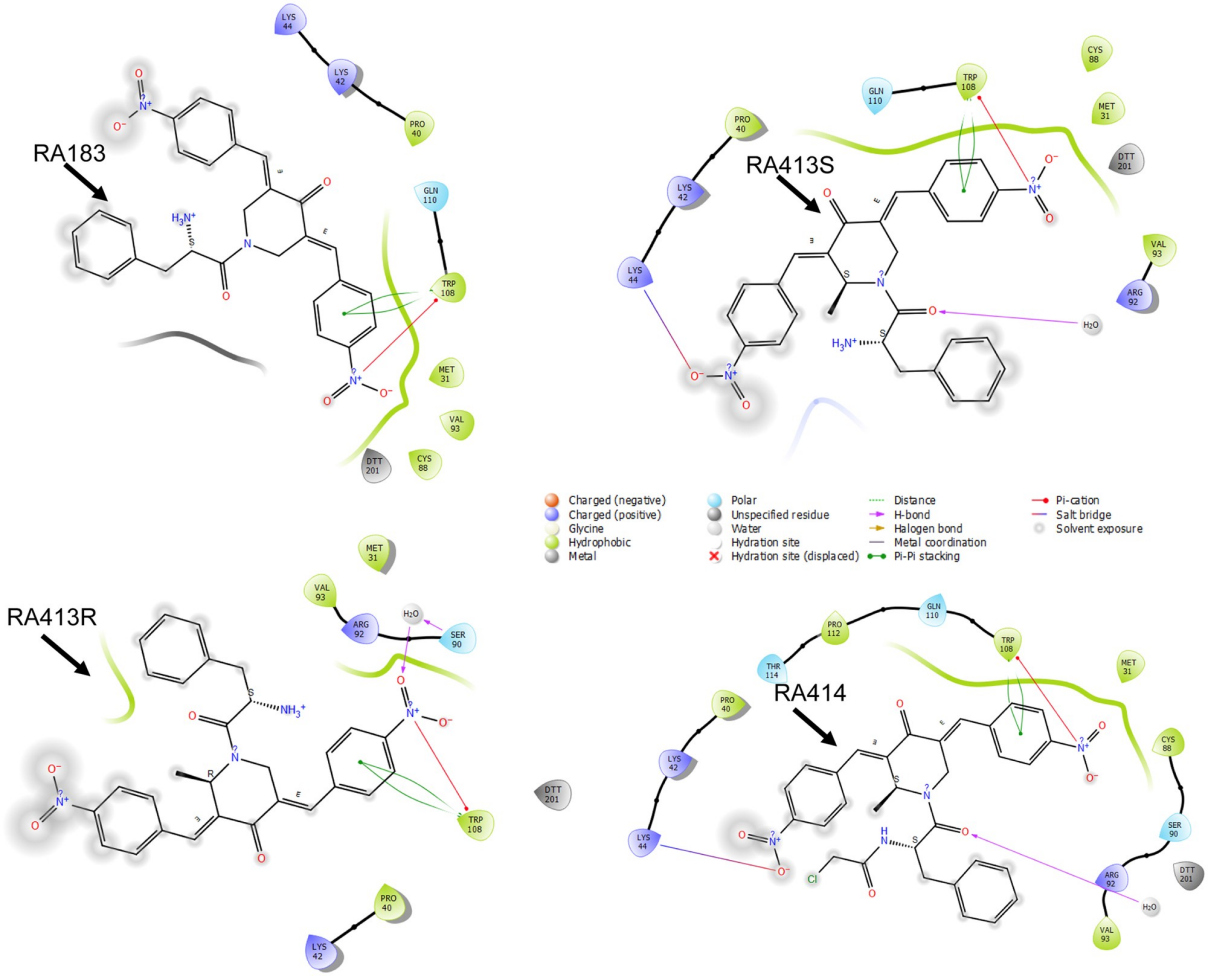
Molecular modeling of compounds binding to RPN13 Pru domain. (A-E) Docked complex of RA molecules (RA183, RA413S, RA413R and RA414) and RPN13 Pru Domain in the Schrödinger glide application.

In our previous studies seeking improved specificity, potency and solubility of our candidate iRPN13s, we generated several libraries of molecules to probe the pharmacophore of the bis-benzylidinepiperidone core unit and identified two active compounds, RA183 and RA375 [11, 14, 15]. Based on these findings and preliminary molecular modelling data, we introduced a methyl group at the ring carbon atom next to the nitrogen of RA183 and RA375 (to create RA413 and RA414 respectively and render them asymmetric and chiral e.g. RA413S and RA413R) and we also examined the impact of electron withdrawing groups within RA413 with *m*,*p* dichloro or *o* or *p* fluoro ring substituents (Fig 1). In the docking studies (Fig 2), RA414 showed a superior Glide Score of -5.43 kcal/mol, with a good binding energy value of (MMGBSA) of -49.69 kcal/mol. RA413S exhibited an intermediate Glide Score of -3.70 kcal/mol and a binding energy value of (MMGBSA) -35.96 kcal/mol. Finally, RA183 had the weakest Glide Score of -2.87 kcal/mol and a binding energy value of (MMGBSA) -34.18 kcal/mol. RA413R had a Glide Score of -3.40 kcal/mol and a binding energy value of (MMGBSA) -39.11 kcal/mol, slightly weaker than seen for RA413S. Cysteine 88 of RPN13 is adducted by RA183. In examining the predicted important contacts within 4Å, cysteine 88 is identified in the model of RA183, RA414, and RA413S, but is not evident for RA413R. This modeling suggested potential for differing RPN13 binding and potencies in cytotoxicity assays of RA414, RA413S and RA183 and for weaker cytotoxicity of the RA413R as compared to its isomer RA413S (Fig 2E).

All these four racemic compounds were tested for cytotoxicity first in HeLa cells (S1 Table). RA413 was similarly potent to its parental molecule RA183 (96 nM vs. 112 nM), but since RA413 is racemic and modeling suggested differential binding, we synthesized each chiral form, RA413S and RA413R (Fig 1B). Interestingly RA413S was 5-fold more cytotoxic for HeLa than RA413R (23 nM vs. 172 nM, S1 Table). This same phenomenon was also apparent in additional cell lines derived from ovarian cancer (e.g. SKOV3, TOV21G)) and cervical cancer (HeLa, CaSki, SiHa) (Table 1 and S1 Table). RA413S was also active against cells lines derived from various histotypes of ovarian cancer (e.g. serous [OVCAR3, SKOV3, UWB1.289, PEO1, PEO14], endometriod [A2780], clear cell carcinoma [TOV21G, ES2] and poorly differentiated adenocarcinoma [PEA1, PEA2]) (Table 1), and an assortment of cell lines derived from other solid malignancies, including colon, breast, prostate and head and neck cancers (S1 Table). Cytotoxicity of RA413S for normal cells (human foreskin fibroblasts (HFF), and prostate epithelial cells (PrEC) was much weaker, with IC50 values >1000 nM, suggesting a useful therapeutic window. We also examined the impact of electron withdrawing groups within RA413 upon in vitro potency, and as described previously the *m*,*p* dichloro or *o* or *p* fluoro ring substituents were less active (Table 1).

**Table 1:** IC50 measurements for candidate iRPN13s in ovarian cancer cell lines (nM). T22, BR5-FvB1, BR5-Luc, C2KmFvB1 and ID8-vegf are murine model lines, and the remainder are of human origin.

Ovarian cancer Cell line name	RA190	RA183	RA375	RA413S	RA413R	RA413	RA414	RA462	RA467	Cisplatin	Olaparib	Bortezomib	Doxorubicin
T22 (BRCA1 wt)	16325			1364									
BR5-FvB1 (BRCA1 null)	250			125						2500	30	<20	<20
BR5-Luc (BRCA1 null)	38			5.7						332	500	4.1	237
C2KmFvB1 (BRCA1 wt)	436			550						7593	7961	3.2	27
A2780	139	161	37	20			13					3	
TOV21G	148	53		3.6	20	42	1.1						
ID8-vegf	211	198	44	60		192	34	123	167			1.5	
SKOV3	73	54	26	40	161	86	19	112	126			1.5	
SKOV3-TR	109	77	22	60			16						
OVCAR3	120	78	18	69			9	105	120	3034		30	
UWB1.289+BRCA1				44.8			<20				994		
UWB1.289 (BRCA1 null)				43.9			<20				581		
PEA1	386			70			8			7519		30	
PEA2	396			64			6			9846		30	
PEO1	232			31			17			7990		31	
PEO4	168			41			12			19470		31	
PEO14	375			92			16			1378		30	
ES2	115	75	19	40			7.5	96	112				

Addition of the chloroacetamide to RA183 produces the more potent analog RA375 [15]. The increase in potency conferred by the chloroacetamide warhead likely reflects soaking up intracellular GSH, as described for RA375 [15]. Likewise, addition of the methyl group to RA375 produced a highly potent racemate, RA414 (Fig 1B), with an IC50 of 3 nM for HeLa cells, compared to 13 nM for RA375 (S1 Table).

### RPN13 binding

**Fig 3:**
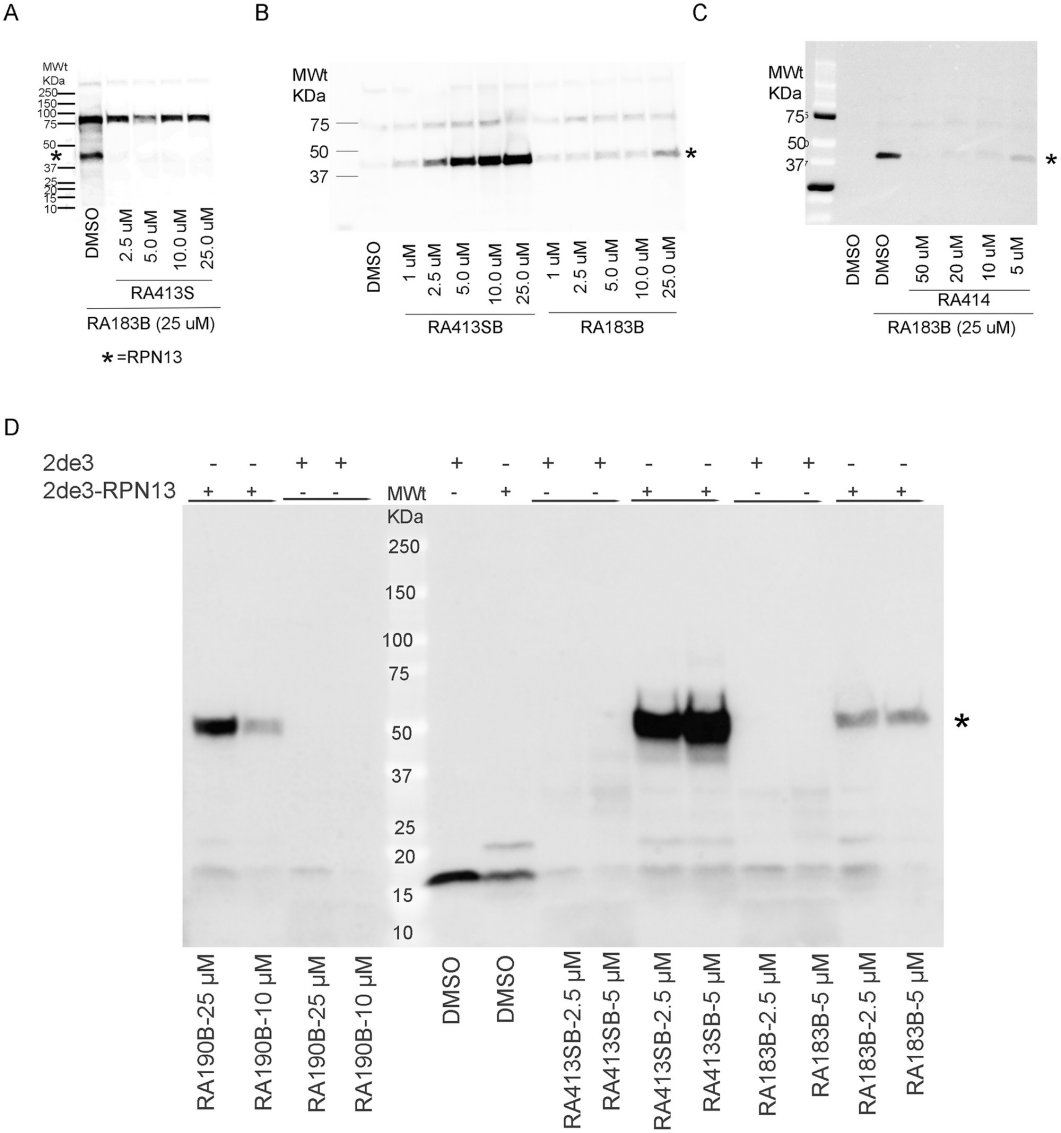
Compounds binding to RPN13 in cell lysate. (A) SKOV3 cell lysate was pretreated with RA413S at indicated concentrations and labelled with RA183B, and (B) ES2 cell lysate was treated with titrations of the biotinylated compound. (C) SKOV3 cell lysate was pretreated with RA414 at various concentrations and labelled with RA183B. (D) Lysate of E. coli 2DE3 overexpressing RPN13 from pET28, or without RPN13 as a negative control, was labelled with 5μM of either RA413SB, RA190B or RA183B. In each case, after treatment the samples were separated by SDS-PAGE, transferred to a PVDF membrane which was probed with HRP-streptavidin to detect biotinylated proteins.

To examine whether RA413S and RA414 bind similarly to RPN13 as their parent compound RA183, we performed a competition assay using biotinylated RA183 (RA183B, see [14]) as a probe (Fig 3A). Briefly, SKOV3 cell lysate was pre-treated with different concentrations of RA413S and then labelled with RA183B. Samples were subjected to SDS-PAGE and transfer to a PVDF membrane. The membrane was probed with horse radish peroxidase (HRP)-linked streptavidin, and the blot developed using chemiluminescence. Imaging revealed that the binding of RA183B to the ~42kDa cellular protein was competed by RA413S in a dose dependent manner, suggesting that they bind in the same manner to RPN13 (Fig 3A) [14].

Biotinylated RA413S (RA413SB) was synthesized and its binding to RPN13 assessed. A direct labelling assay in cell lysate of ES2, a human ovarian clear cell carcinoma-derived cell line, was performed using RA413B and RA183B. Binding of RA413SB to the 42kDa cellular target was substantially stronger than for RA183B, consistent with the higher potency of RA413S than RA183 in cytotoxicity assays (Fig 3B). Binding of RA183B to the 42kDa cellular protein is inhibited by pre-incubation with RA414, suggesting that it also adducts Cysteine 88 of RPN13 (Fig 3C) [14]. Similarly, labeling of 2DE3 bacterial cell lysate expressing RPN13 from pET28 (tagged with 6His and T7tag), or not as a control, demonstrated stronger RPN13-specific reactivity with RA413SB compared to RA183B, which was also more robust than for RA190B, as previously described (Fig 3D).

### Impact on proteasome function

**Fig 4:**
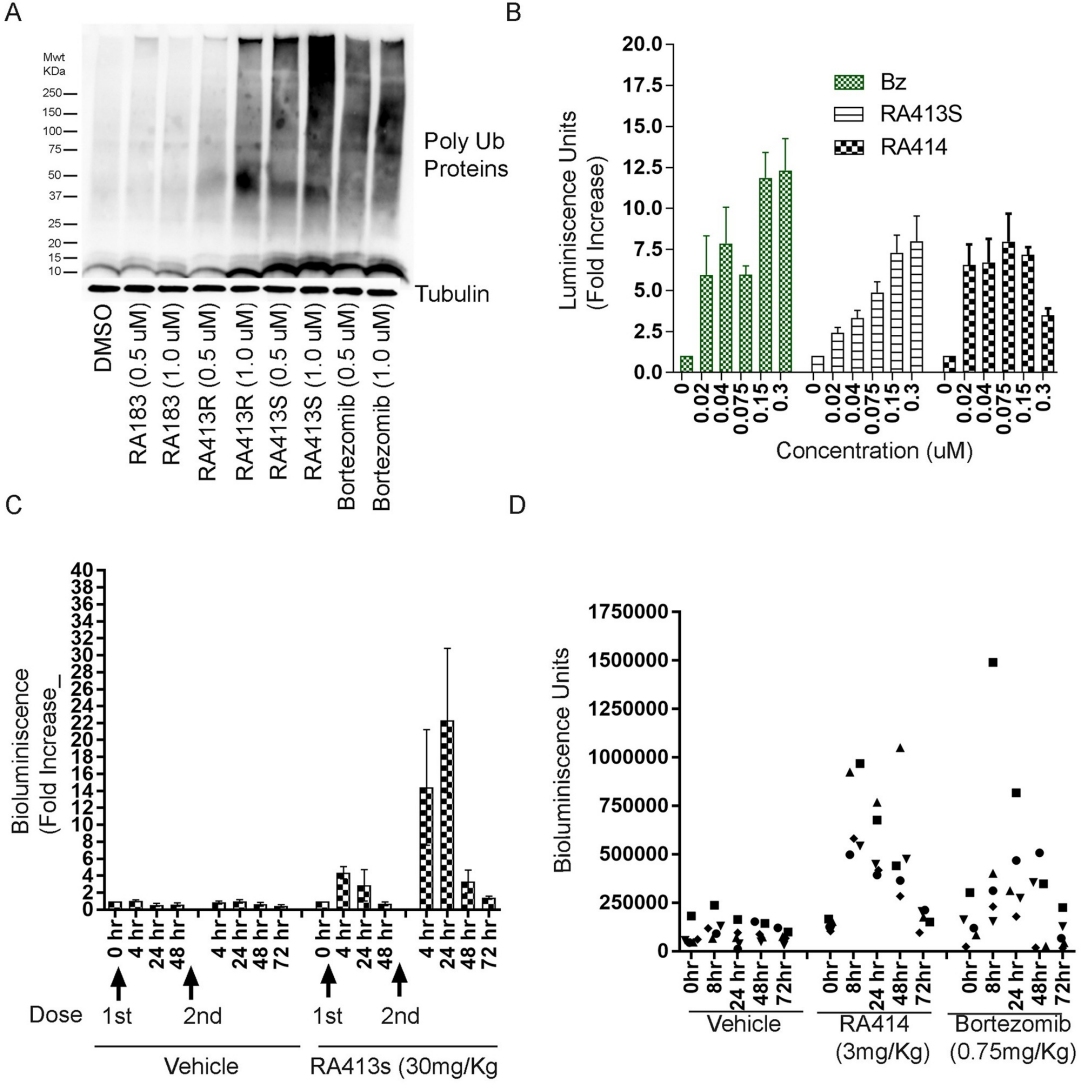
Compounds inhibit proteasome function in vitro and vivo. (A) SKOV3 cells were treated with compounds for 4 h and analyzed by Western blot with antibody to ubiquitin to assess the accumulation of high molecular polyubiquitinated proteins associated with proteasomal inhibition. (B) Fold increase in bioluminiscence of ES2 cells stably expressing 4UbFL at 4 h post-treatment of compounds, as measured by luminometer. (C & D) Leg muscle was electroporated after i.m. injection with 4UbFL plasmid. 3 days later IVIS200 imaging was performed upon injection of D-luciferin to assess the bioluminescence of female CD1 mice leg muscle before (0 h) and 4, 24, 48, and 72h after drug treatment. Increased bioluminescence was observed after treatment with RA413S; the first dose utilized PEG formulation, and the second dose 72 h later used a b-HPCD formulation. (C, figure showing fold increase) or RA414 or bortezomib (D, figure showing bioluminescence units for individual mice).

We examined the effect of RA413S and RA414 on proteasome function in ovarian cancer cell lines in comparison with the previously described RA183, RA190, RA375 and the licensed 20S PI bortezomib. All enhanced the accumulation of polyubiquitinated proteins in SKOV3 cells (Fig 4A). Treatment of SKOV3 with the candidate RPN13 inhibitors enhanced the accumulation of the highest molecular weight polyubiqitinated proteins that did not fully enter the gel. Notably, RA413S was more potent compared to its R isomer and parent compound RA183 in triggering this accumulation (Fig 4A). Similar results were obtained with RA413S and RA414 in HeLa cells derived from a human cervical cancer, and in the human colon cancer cell line HCT116 (S1A and S1B Fig).

IκBα ubiquitination and proteasomal degradation is an important step for the nuclear localization of NFκB and activation of its transcriptional program. We previously observed that RA183 inhibits this signaling pathway with an IC50 of 0.1 μM [14] as determined using 293T cells transfected with an NFκB-responsive luciferase reporter construct. Using the same system, RA413S demonstrated a dose dependent inhibition of in NFκB-dependent luciferase activity that was more potent than for RA183, but less potent than RA414 (S1C Fig).

Next we examined the effect of compounds on proteasome function using a DNA construct that expresses a reporter gene comprising firefly luciferase fused in tandem to four molecules of ubiquitin (4UbFL) at its amino terminus [21]. The 4UbFL is rapidly degraded by active proteasomes, resulting in a low level of enzymatic activity and bioluminescence. The 4UbFL protein is stabilized by proteasome inhibition this can be directly measured as increased bioluminescence using D-luciferin substrate. For ES2 cells stably expressing 4UbFL treatment with either RA413S or RA414 increased bioluminescence in a dose dependent manner. The concentration dependence was consistent with the IC50 of the compounds, but was measured at 4 h, well before the onset of cell death (Fig 4B).

We performed pilot safety studies in CD1 mice (n = 3/dose group of healthy female mice aged 6–8 weeks) with increasing single doses delivered i.p. and selected the NOAEL for further animal studies. Intra peritoneal administration of RA413S every 3 days for 3 doses was tolerated at 30mg/Kg without weight loss or any other clinically noticeable toxicities, whereas the NOAEL for RA414 when administered every 3 days for 3 doses was 3mg/Kg. Bortezomib was dissolved in a minimal amount of DMSO and further diluted with saline before injection in a dose finding study. An NOAEL of 0.75 mg/Kg was identified for bortezomib via this route every 3 days for 3 doses. To measure on-target inhibition of proteasome activity in mice, we transduced a leg muscle of CD-1 mice with the 4UbFL reporter construct. The time course of stabilization of 4UbFL expressed in the muscle tissue after delivery of a single dose of RA413S, RA414, or bortezomib by i.p. injection was followed using an IVIS200 imager. Briefly, mouse leg muscle was electroporated immediately after injection with 4UbFL plasmid, and basal bioluminescence was measured after 24 h using IVIS200 imager after injecting luciferin substrate. Mice were randomized (n = 5/group) and treated with compounds in PEG400: Cremophor: Tween40 (PCT) excipient or excipient alone as shown in Table 2 with one dose delivered by intra peritoneal injection. The mice were again imaged at 4 h, 24 h, 48 h post treatment. RA413S treatment group exhibited 4–6 fold increased bioluminescence at 4 h and 24 h but returned to basal levels by 48h. At 72 h, another dose of RA413S formulated in b-HPCD was administered by i.p. injection, and the mice were again imaged at 4, 24, 48, and 72 h. RA413S increased the bioluminescence by 14–22 fold at 4 h and 24 h later, and this returned to the basal level by 72 h. The data indicates on-target activity of the compounds in vivo. RA413S formulated in b-HPCD (β-hydroxy propyl cyclodextrin) shows higher and longer on-target activity compared to the PCT formulation (Fig 4C). Based on these observations we selected the b-HPCD formulation for RA413S. RA414 was soluble in the PCT system only, but it possessed on-target activity over 72 h similar to bortezomib (Fig 4D). These data suggest an every third day dosing regimen is appropriate for RA413S, RA414 and bortezomib upon intra peritoneal delivery.

**Table 2:** Dosing regimen of compounds and IVIS imaging schedule. 5 CD1 mice were imaged per compound. (bHPCD = β-hydroxypropyl cyclodextrin).

Compound	1^st^ dose (i.p.)	Formulation	IVIS imaging schedule (hours)	2^nd^ dose (i.p.)	Formulation	IVIS imaging schedule (hours)
Vehicle		PEG400:Cremophor:Tween40	0, 4, 24, 48		25% bHPCD	0, 4, 24, 48, 72
RA413S	30mg/Kg	PEG400:Cremophor:Tween40	0, 4, 24, 48	30mg/Kg	25% bHPCD	0, 4, 24, 48, 72
RA414	3mg/Kg	PEG400:Cremophor:Tween40	0, 4, 24, 48, 72			
Bortezomib	0.75mg/Kg	DMSO and Saline	0, 4, 24, 48, 72			

### RA413S impacts mitochondrial function

**Fig 5:**
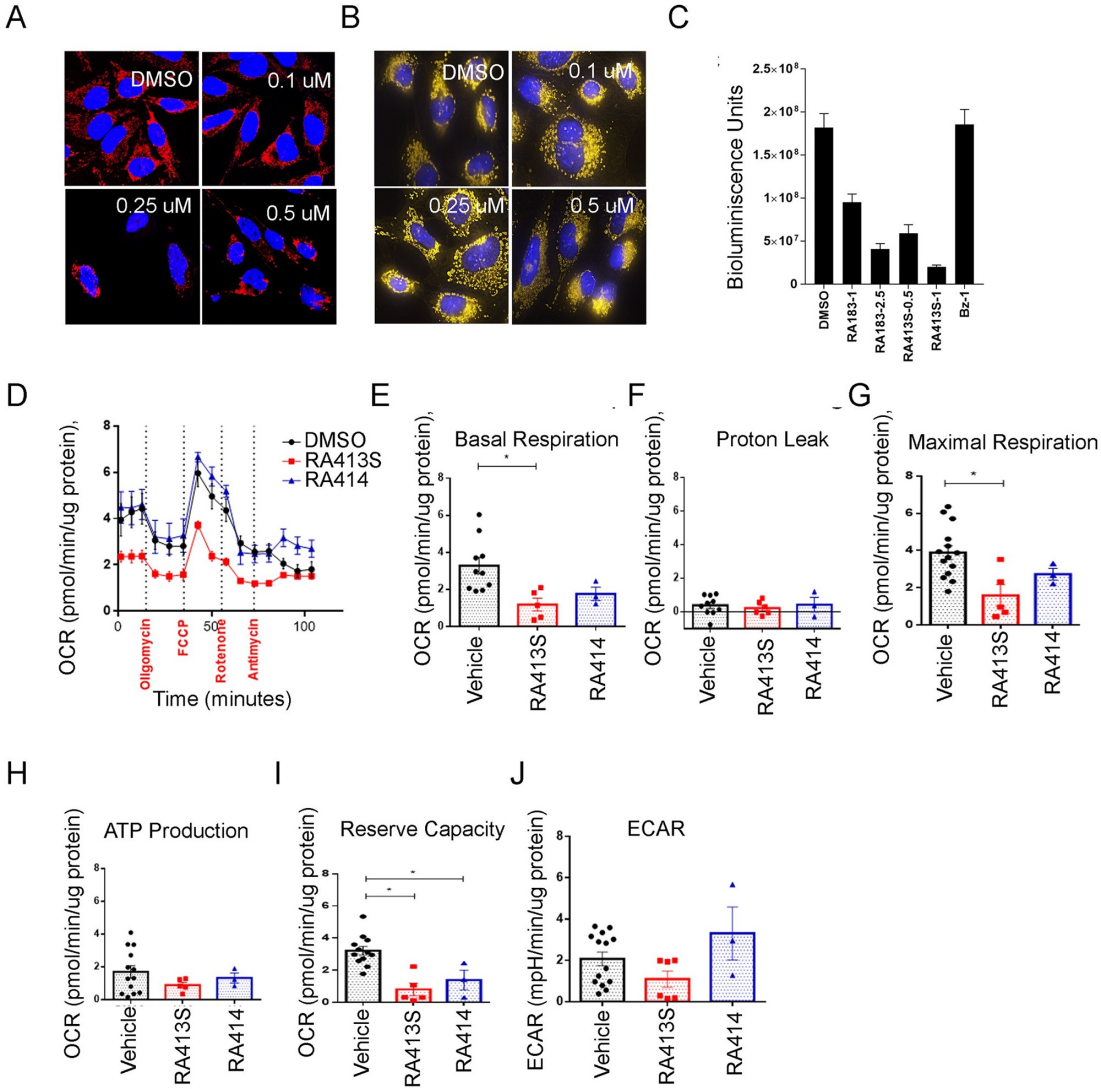
RA413S induces mitochondrial damage and alters cellular bioenergetics. Fluorescent microscopy images of (A) HeLa (B) BR5 cells treated with RA413S for 18 h at the indicated concentrations. Cells were fixed and stained with MitoTracker (red [A]/yellow [B] pseudocoloring and Hoechst (blue). Fragmented mitochondria is visible in RA413S treated samples (C) Assessment of the impact of treatment upon intracellular ATP levels using bioluminescence of ES2 cells expressing firefly luciferase after addition of D-luciferin; RA183 and RA413S treatment caused reduction ATP-dependent luminescence. (D-I) Seahorse assay in ES2 cells upon treatment with RA413S (100 nM) and RA414 (100 nM) or vehicle (DMSO) for 12 h. (D-J) Oxygen consumption and extracellular acidification rates by ES2 cells upon treatment with 500 nM of RA413S and 100 nM RA414 or vehicle (DMSO) for 12 hr. Mitochondrial function was measured following sequential additions of oligomycin, FCCP, Rotenone and Antimycin A into the culture media. (E-J) Oxygen consumption rate (OCR) in ES2 cells as measured by a Seahorse XF Analyzer. Data are representative of 3 independent experiments, and 9 biologic replicates per conditions were used.

Bortezomib reduces mitochondrial membrane potential, PINK1/Parkin expression levels, and caused perinuclear clustering of mitochondria, yet it changes neither mitochondrial dynamics nor mitophagy rate [22]. Parkin plays an important role in regulating the clearance of mitochondrial proteins during mitophagy and this mitochondrial E3 ubiquitin ligase is recruited to the proteasome via RPN13 [23], suggesting mitochondrial function may be impacted by iRPN13. Furthermore, GSH must be imported into mitochondria because they cannot synthesize it [24], and maintaining adequate mitochondrial GSH levels is necessary to scavenge ROS produced during mitochondrial metabolism and prevent apoptosis [25]. MitoTracker is a fluorescent dye that covalently labels mitochondria within live cells utilizing the mitochondrial membrane potential. Using Mitotracker to stain HeLa cells after RA413S treatment for 18 h, mitochondrial depolarization was evident over time as a weaker signal visualized by fluorescent microscopy (Fig 5A) as well as the perinuclear clustering of mitochondria previously seen with bortezomib [22]. Similar results were obtained with RA413S treatment in a murine ovarian cancer model cell line BR5 [26] although the effects were less prominent (Fig 5B), consistent with its lower sensitivity to RA413S (Table 1).

Because of these effects upon mitochondrial morphology and membrane potential, the impact of RA183 and RA413S treatment on overall cellular ATP production were analyzed using a luciferase-based assay. Briefly, firefly luciferase expressing ES2 cells were treated for 4 h with compounds at sub lethal doses and the bioluminescence was measured in intact cells using luminometer by adding D-luciferin. We observed a reduced bioluminescence with RA183 and RA413S treatment indicating reduced intracellular ATP levels. This was not evident with bortezomib treatment (Fig 5C) which is surprising as it produces mitotoxicity in neuronal cells by reducing ATP production [27]. However bortezomib also enhances ATP production by glycolysis which may compensate for suppressing oxidative phosphorylation (OXPHOS) [28].

To examine the mechanism underlying this rapid drop in intracellular ATP cells (Fig 5C), we examined in ES2 cells whether a reduction in both oxidative phosphorylation (OXPHOS), and glycolysis occurred with RA413S or RA414 treatment (Fig 5D–5J). Mitochondrial electron transport chain (ETC) dysfunction, oxygen consumption rate (OCR) and anaerobic acid production via extracellular acidification rate (ECAR) were assessed in intact ES2 cells using a Seahorse XF flux analyzer. OCR was measured at baseline and after addition of oligomycin, a complex V inhibitor, followed by an uncoupler, carbonilcyanide p‐triflouromethoxyphenylhydrazone (FCCP), then rotenone (complex I inhibitor), and finally antimycin (complex III inhibitor) (Fig 5G). Upon 12 h of RA413S treatment there was a significant decrease in OCR, both in basal (Fig 5E) and respiration stimulated with FCCP (Fig 5G). However, the OCR suppression with RA414 did not reach significance. The overall OXPHOS inhibition with RA413S and RA414 treatment significantly reduced the mitochondrial energy reserve in ES2 cells, which is essential for cell survival (Fig 5I). Furthermore, both RA413S and RA414 treatment showed a decrease in mitochondrial ATP production by inhibiting the OXPHOS (Fig 5H). A trend for reduction in ECAR, a surrogate measure of glycolysis, was observed after 24 h RA413S treatment (Fig 5J). These observations suggest that RA413S affects ES2 cancer cells by inhibition of glycolysis and OXPHOS, thereby ultimately reducing mitochondrial ATP production. Similar patterns were observed in ES2 cells with the treatment of RA183 (S2 Fig), suggesting that it also acts like RA413S to starve the cancer cells of their energy source, but RA414 may not affect OXPHOS.

### RPN13 inhibitors trigger oxidative stress and apoptosis

**Fig 6:**
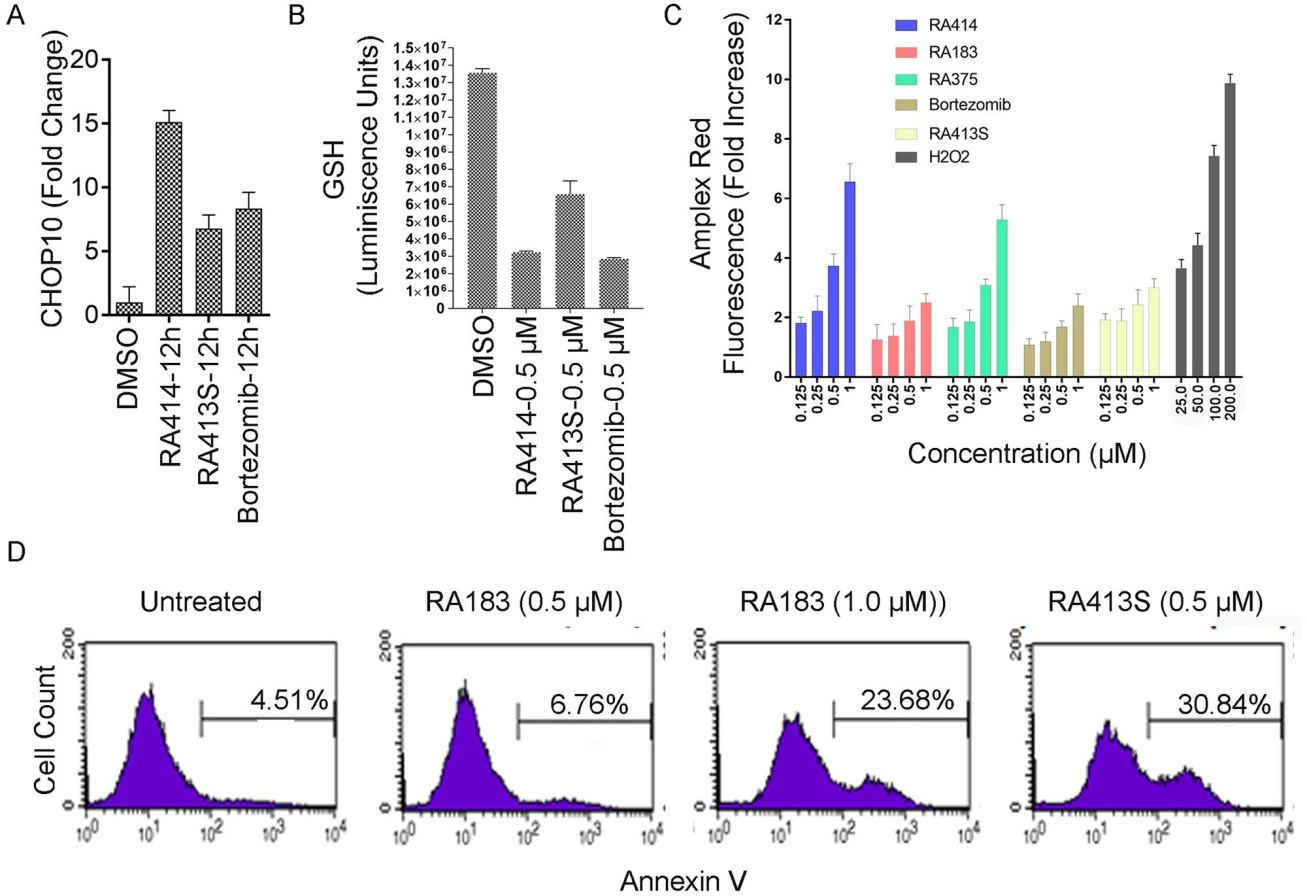
RA413S and RA414 induce both ER and oxidative stress triggering apoptosis. (A) ES2 cells treated with compounds (0.5 μM) for 12 h and CHOP-10 mRNA levels, an ER stress marker, was analyzed by qRT-PCR. (B) ES2 cells were treated with compounds for 12 h and the total GSH level was assayed. (C) ES2 cells treated for 12 h with compounds (or as a positive control, H_2_O_2_) at the indicated doses, and then ROS levels were measured by adding Amplex Red and HRP. (D) To examine the early onset of apoptosis, ES2 cells were treated with compounds at indicated doses for 12 h then re-suspended in 100 μL binding buffer with 5 μL of Annexin V-PE and 5 μL of 7-AAD. After a 15 min incubation at RT, the cells were analyzed by flow cytometry using a FACSCalibur and CellQuest software.

Recent insights indicate that ER stress and oxidative stress are highly interrelated cellular processes which regulate a wide range of signaling pathways. ROS generation plays a critical role in initiating proteasome inhibitor-induced apoptosis [29, 30]. To counteract ROS, cells upregulate antioxidant pathways including intracellular GSH. GSH is the most abundant intra cellular non-protein thiol and it can reduce the potency of iRPN13s. Conversely, iRPN13s reduce GSH upregulation and increase cellular ROS [15]. Thus the impact of RA413S and RA414 on an ER stress marker, CHOP10 mRNA, was determined by RT-PCR, GSH levels using a fluorometric GSH assay kit, ROS by Amplex Red assay and as a biomarker of early apoptosis, surface Annexin V staining was measured by flow cytometry. As expected RA183, RA413S and RA414 each significantly increased CHOP10 transcript levels (Fig 6A), reduced intracellular GSH concentrations (Fig 6B), raised the level of ROS in a dose-dependent manner (Fig 6C) and triggered apoptosis (Fig 6D). In sum, these findings indicate that the induction of cellular and mitochondrial ROS upon RA413S, RA183 or RA414 treatment contributes to their cytotoxicity. Similar results were observed with these compounds in another ovarian cancer cell line, SKOV3 (S3 Fig).

### Therapeutic efficacy of RA413S and RA414 against syngeneic and xenograft mouse models of ovarian cancer

We sought to determine the maximum tolerated dose (MTD) of a single administration of either RA413S or RA414 in groups of 3 healthy female CD1 mice (4–6 weeks old) per dose level using >10% weight loss and/or the emergence of clinically unacceptable symptoms as end points. The starting doses were based on previous studies with RA183. Doses were escalated incrementally in steps of not more than 50%. When either of these endpoints was met, dose escalation was halted and the prior dose was set as the MTD. Next, we determined the effect of 3 repeated doses of each compound every third day in healthy female CD1 mice and identified repeat dose MTDs of 20 mg/kg for RA413S and 10 mg/kg for RA414.

**Fig 7:**
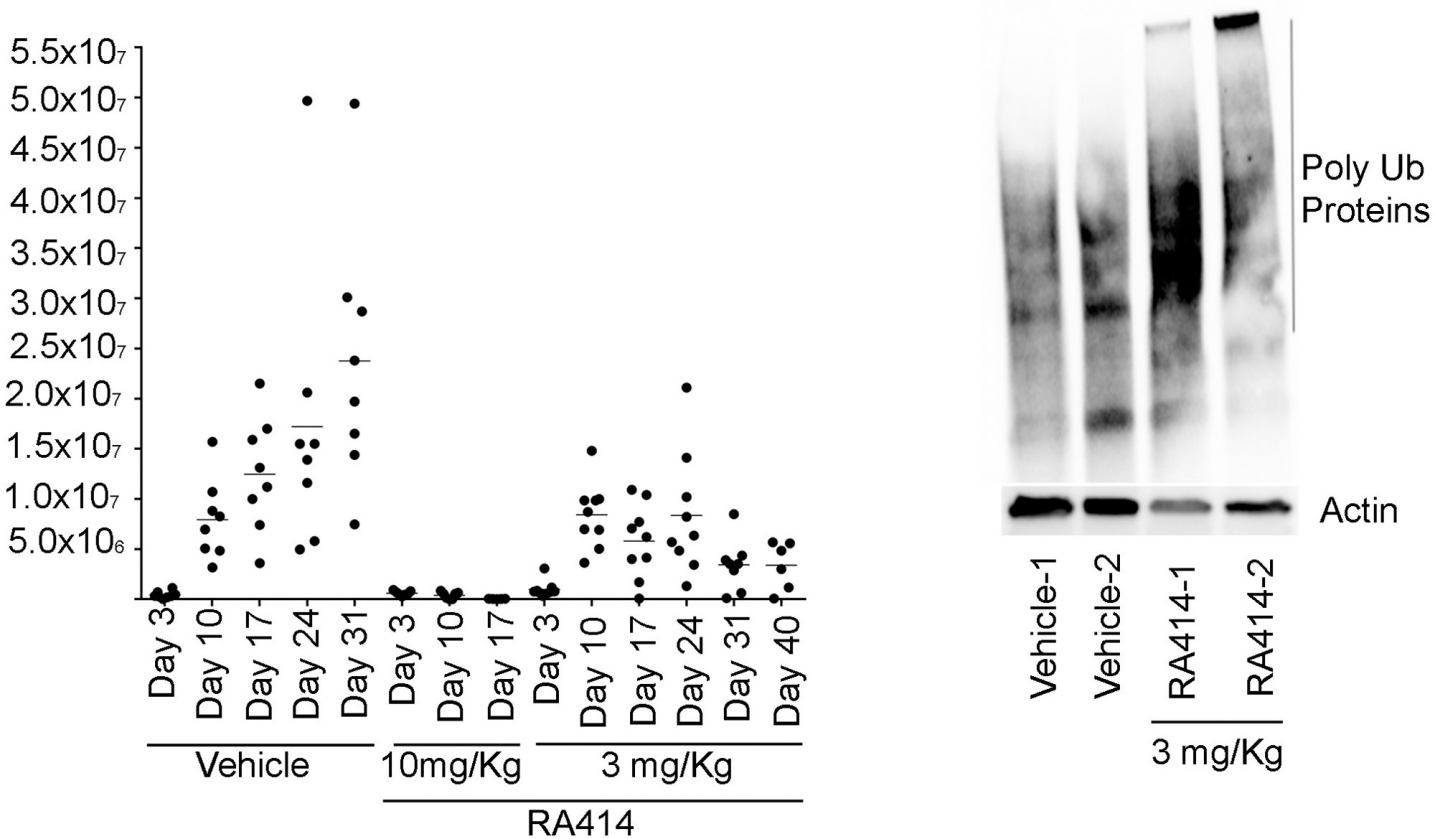
Tumor growth inhibition and on-target effect of RA414 in a syngeneic mouse model of ovarian cancer. (A) C57BL/6 mice bearing intraperitoneal ID8-Vegf+ *Defb29+* tumor that expresses luciferase was imaged using an IVIS200 at the indicated days following treatment with either vehicle (PEG400: Cremophor: Tween40) or RA414 (10 mg/Kg/every 3 days/ 3 doses total) and RA414 (3mg/Kg/every 3 days/total 9 doses). (B) From Fig A, two mice each from the vehicle and RA414 (3 mg/Kg) treated mice were euthanized and ascites collected at day 24. The ascites cells were subjected to Western blotting analysis using antibody to ubiquitin or, as a loading control, actin.

To assess therapeutic efficacy we utilized the ID8-Vegf *Defb29* cell line, a syngeneic mouse model of ovarian cancer expressing luciferase [20]. ID8 was first derived from spontaneous malignant transformation of C57BL/6 mouse ovarian surface epithelial cells in vitro [31]. ID8-Vegf *Defb29* cells represents more aggressive variant of ovarian cancer in which VEGF provides vascular growth which is augmented by the presence of β defensin 29 [20]. C57BL/6 mice were injected i.p. with ID8-Vegf *Defb29* cells stably expressing firefly luciferase (0.5X10^6^ cells) in PBS (100 μL). After 72 h, mice were imaged for their basal luminescence levels using an IVIS200 imager. Mice with visible luminescence were randomized into equal groups to receive vehicle (n = 8), RA414 (3 mg/Kg, n = 8), or RA414 (10mg/Kg, n = 8) treatments every three days for up to 3 weeks. Mice were imaged every week to measure bioluminescence expressed by tumor after administration of luciferin. In the vehicle group, all 8 mice showed enlarged abdomens due to ascites formation. On day 24 two mice required euthanasia and were sacrificed after collection of ascites, and the remainder required euthanasia on day 31 (Fig 7A and 7B). Administration of RA414 at 10mg/Kg (3 doses delivered every third day) produced complete responses in 50% of the mice with less than 10% weight loss. However, the remaining 50% of the mice did not tolerate the dose (>10% weight loss) or died, indicating this is above the MTD in tumor-bearing mice. Administration of RA414 at 3 mg/Kg (9 doses delivered every three days) was well tolerated by the mice and inhibited tumor growth (Fig 7A). Furthermore, analysis of ascites cells collected on day 24 from two RA414-treated mice by Western blotting with antibody to ubiquitin demonstrated the accumulation of high molecular polyubiquitinated proteins that was absent from ascites of control mice (Fig 7B). This is consistent with proteasome inhibition within the ascites cells by the 3 mg/Kg RA414 treatment regimen.

**Fig 8:**
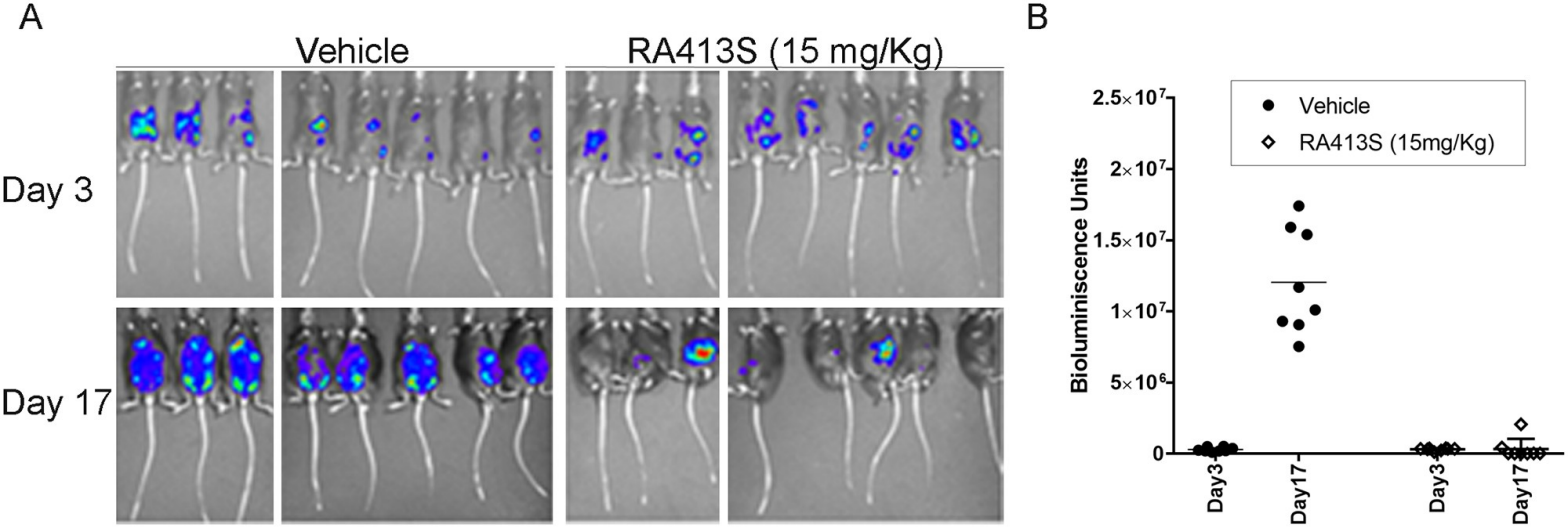
Tumor growth inhibition by RA413S in a syngeneic mouse model of ovarian cancer. (A) C57BL/6 mice administered ID8-Vegf+ *Defb29+* syngeneic mouse model of ovarian cancer expressing luciferase [20] intraperitoneally were imaged for randomization by tumor burden before treatment (at day 3 post tumor inoculation, n = 16). Treatment was initiated on day 3 unblinded and the mice were imaged again after treatment with either vehicle (25% β-hydroxypropylcyclodextrin) or RA413S (15mg/Kg/every 3 days/total 5 doses) in 8 mice per each group on day 17 using IVIS200 to quantify the bioluminescence expressed by the tumor cells. Bioluminescence at days 3 and 17 is presented for all mice (B). A significant difference in bioluminescence was observed p<0.0001.

To examine the in vivo activity of RA413S, female C57BL/6 mice were injected i.p. with the ID8-Vegf *Defb29* syngeneic mouse cell line model of ovarian cancer expressing luciferase [20] and imaged by IVIS200 on day 3 just before treatment to ensure tumor take and for randomization (Fig 8A). Treatment was initiated on day 3 and the mice were imaged again after treatment with either vehicle or RA413S (15mg/Kg, every 3 days for a total of 5 doses) on day 17 using IVIS200 imaging to quantify the bioluminescence expressed by the tumor cells (Fig 8A and 8B). This treatment produced a significant decrease in tumor burden compared to vehicle (p<0.0001).

**Fig 9:**
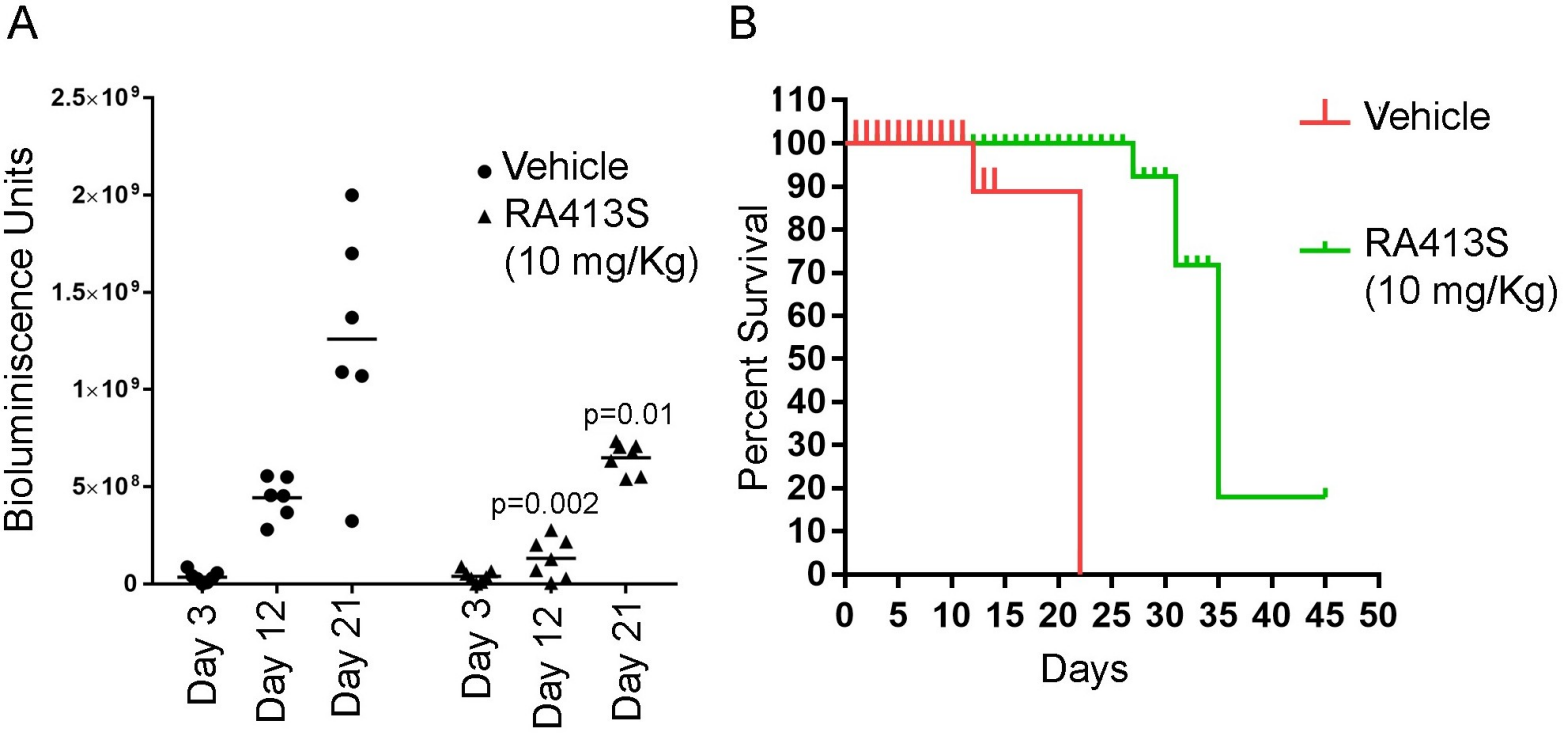
Tumor growth inhibition effect of RA413S against ovarian cancer xenograft. (A) Female nude mice bearing ES2 tumor expressing firefly luciferase (n = 14) were imaged before and after unblinded treatments either with vehicle (25% β-hydroxypropyl cyclodextrin in water, n = 7) or RA413S (n = 7, 10mg/Kg/every 3 days/12 doses total) and imaged using IVIS200 for the bioluminescence expressed by the tumors. One mouse in the vehicle group died on day 12 and was not included for the day 12 and 21 time points. (B) Kaplan-Meier survival curve and log rank analysis demonstrates nude mice treated with RA413S have extended survival time compared to the vehicle treated mice (p<0.001).

With these promising results, we assessed the efficacy of RA413S against a human ovarian cancer xenograft model. Briefly, female nude mice were injected i.p. with ES2-luc cells (1X10^6^) in 100 μL PBS and the basal luminescence was measured after three days using IVIS 200 imaging after injecting luciferin. Tumor-bearing mice were randomized into two groups to receive either vehicle alone (n = 7) or RA413S (n = 7, 10 mg/Kg, every 3 days for a total of 12 doses). Over the treatment period mice were imaged every week for bioluminescence to monitor tumor growth. Mice treated with RA413S tolerated the drug and showed significantly reduced tumor burden (Fig 9A) and significantly extended survival (Fig 9B).

### Synergistic activity of iRPN13 with cisplatin and doxorubicin

BRCAness has been associated with increased sensitivity to proteasome inhibition and bortezomib [32, 33], and we previously observed that ARID1A loss was associated with increased sensitivity to iRPN13 [15]. Interestingly, isogenic DLD1 cell lines with defective DNA repair pathways due to BRCA2 [34] or ATR loss [35] were more sensitive to RA413S or bortezomib than the parental line, and also, as expected for cisplatin (Table 1). Likewise, ATM-deficient RPE1 cells were more sensitive to both RA413S and bortezomib than the parental line, although the effect with cisplatin was greater. However, the BRCA1 deficient UWB1.289 cell line was similarly sensitive to RA413S after complementation with wild type BRCA1 [36]. This may reflect the previously described only partial restoration of the DNA damage response pathway [36], and indeed only a two-fold increase in the IC50 for cisplatin was observed (Table 1). By contrast, in murine ovarian cancer model cell lines [26], BRCA1 knockout cell line BR was more sensitive to both RA413S and cisplatin that BRCA1 wild type lines T22 and C2 (Table 1).

Platinum agents like cisplatin or carboplatin are the primary therapeutic option for ovarian cancer patients, and have shown promise in combination with *i*.*p*. bortezomib in an early phase clinical study [37]. They produce DNA damage and activate the DNA damage repair pathway (DDR) in a ubiquitin-dependent manner [38]. Proteasome inhibition increases sensitivity to DNA damage by blocking repair [39] and we hypothesized that the depletion/sequestration of mono-ubiquitin levels by iRPN13 (S1A Fig) may likewise interrupt the DNA damage response pathway and enhance sensitivity to cisplatin (S4A–S4D Fig). Therefore, we examined whether cisplatin is synergistic with an iRPN13. Combination treatment with RA414 and cisplatin showed evidence of synergy in cytotoxicity of OVCAR3 cells (S4E–S4H Fig) when the combination effect was analyzed using a Synergy finder web application to determine the ZIP score [40]. Similarly RA413S and RA414 demonstrated strongly positive ZIP scores when combined with cisplatin or doxorubicin, agents used in first and second line treatment of ovarian cancer, in various ovarian cancer cell lines suggesting synergistic combinations (S4 Fig).

## Conclusions

Targeting the 19S RP with non-peptide based small molecules is a promising approach to overcome the resistance and toxicities associated with currently licensed 20S CP inhibitors, and improve access to, and thereby activity against solid tumors. A number of small molecules have been developed as candidate inhibitors of several subunits of the 19S RP including ubiquitin receptors RPN10 and RPN13, deubiquitinases USP14, UCH37 and RPN11, and ATPase RPT4 (reviewed in [41]). USP14 has been targeted with IU1 [42] and IU1-248 [43], as well as by b-AP15 [1] and VLX-1570 [2] that also bind UCH37 [3]. RPN11 inhibitor capzimin [44] and RPT4-binding peptoid RIP-1 were also identified [45] but their activity in vivo is not known. In addition to our molecular series [11, 14, 15], RPN13 has been targeted with siRNA [12], diphenyldihaloketones CLEFMA and EF24 [46], peptoid approaches [47], thalidomide-based degrader WL40 [48] and, interestingly, Shigella flexneri invasion plasmid antigen H (IpaH) 4.5 is a E3 ubiquitin ligase that induces RPN13 degradation via the proteasome and enhances bacterial pathogenicity [49]. However, identifying 19S RP inhibitors with drug-like properties remains challenging and to date only VLX-1570 has been tested clinically [7].

In seeking to improve the specificity and potency of our candidate iRPN13s based on the bis-benzylidinepiperidone core unit [11, 14, 15], we introduced a methyl group at the ring carbon atom next to the nitrogen of a lead compounds from our library, RA183, to create RA413 respectively and render it asymmetric and chiral. Interestingly, the S isomer of RA413 was more active than the R form. Introduction of the methyl group in the same position in RA375 also enhanced potency against cancer cell lines by the resultant RA414. Treatment of ovarian cancer cells with these candidate iRPN13 (RA413S and RA414) caused a rapid build-up of high molecular weight polyubiquitinated proteins of greater size and rapidity than seen with bortezomib.

Treatment with RA413S and RA414 inhibited tumor growth in both syngeneic and xenograft models of ovarian cancer and increased overall survival, and appear more active than RA183 based on prior studies, although head-to-head comparison is warranted. RA413S and RA414 also both show stronger binding to their 42kDa protein target, previously identified as RPN13, in ovarian cancer cell lysates than RA183. Although these reactive compounds have potential for off-target effects [50], RA190 and related inhibitors bind to cysteine 88 within a groove of RPN13 that mediates binding to the proteasome via RPN2 [16, 17]. This suggests that drug blockade of RPN13 association to the 19S RP inhibits both proteasomal degradation and deubiquitinase activity of its binding partner UCH37. In the 19S RP, three deubiquitinating enzymes (UCH37, USP14 and RPN11) disassemble ubiquitin chains prior to substrate transfer to, and degradation in the 20S CP, making them central in the regulation of ubiquitin-mediated degradation. While RPN11 cleaves the polyubiquitin chain at its base where it is linked to the substrate, UCH37 and USP14 progressively remove ubiquitin from the ubiquitin chain from its far end [51]. When RPN13 docks to RPN2 on the 19S RP [52], it both activates ubiquitin binding and recruits UCH37 to proteasome. The binding of UCH37 to RPN13 on the 19S RP also greatly enhances the UCH37’s DUB activity [53–55]. Therefore, an RPN13 inhibitor would potentially prevent activation of UCH37 DUB activity, and produce a buildup of higher molecular weight polyubiquitinated proteins (i.e. aggregates containing more ubiquitin, and longer chains thereof) than seen with 20S proteasome inhibitors which inhibit the substrate degradation but not the proteasome-associated deubiquitinase activity. Indeed, we have observed this phenomenon with our lead RPN13 inhibitor, RA190 [11] and the compounds herein.

**Fig 10:**
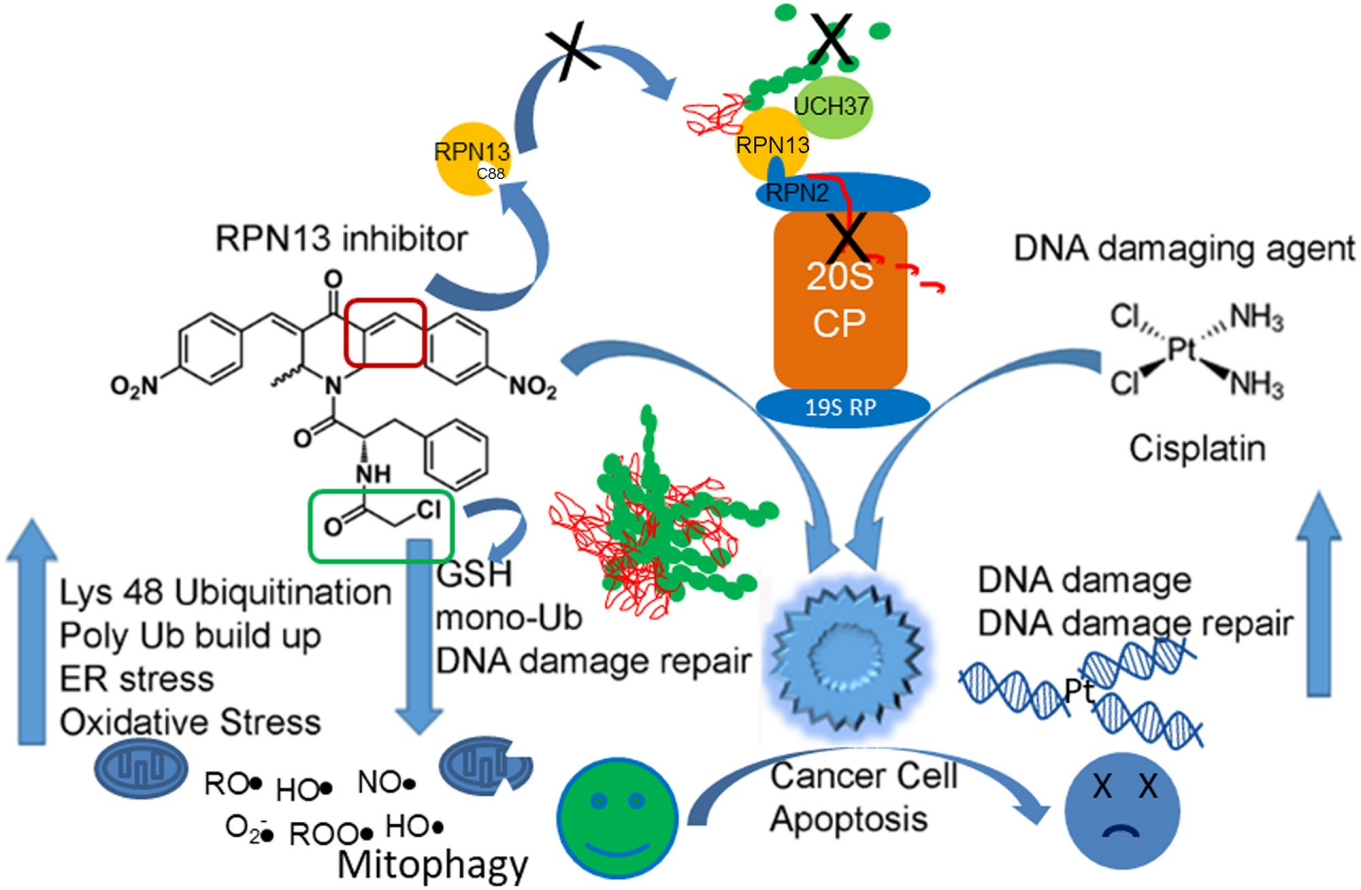
Scheme for the proposed mechanism of action of RPN13 inhibitors and synergistic cytotoxicity observed in combination with DNA damaging agents like cisplatin.

RA413S and RA414 inhibit proteasome function and promote ER stress, oxidative stress, mitochondrial damage, ATP depletion, NF-κB inhibition and finally activate apoptosis in cancer cells more potently than RA183 (summarized in Fig 10). While treatment with these compounds regressed tumor growth and increased survival in mouse models of ovarian cancer, the higher dose of RA414 produced toxicities in tumor-bearing animals suggesting 3mg/kg is its MTD, and that they may be more sensitive to treatment-related toxicity than naïve mice. An initial analysis of RA414 identified synergistic dose combinations with cisplatin suggesting that this might be a potent combination that could allow for the use of lower RA414 doses. We speculate that this may reflect reduced repair of the platinum-induced DNA lesions because of the importance of ubiquitination, and thus free ubiquitin levels, in control of these DNA damage response pathways (Fig 10). RA413S was better tolerated by the tumor-bearing mice. Further studies are warranted to address the involvement of RPN13 in cisplatin resistance, potential for greater activity of these candidate RPN13 inhibitors against tumor with compromised DNA damage responses, and the underlying mechanism of their synergy with cisplatin.

We propose a model in which the inhibitor RA414 (and RA413S) adducts RPN13 via Cys88, but this is competed by cellular glutathione. The drug covalently attached to Cys88 blocks the binding of RPN2 to RPN13 which is essential for tethering to the proteasome and RPN13’s role in recognition and enhancing deubiquitination by UCH37 of polyubiquitinated proteins that are the substrates for proteasomal degradation. As a consequence, misfolded and very high molecular weight polyubiquitinated protein aggregates rapidly accumulate to toxic levels within the cell, and also mop up the pool of available monomeric ubiquitin. These aggregates trigger an unresolved ER stress response which promotes apoptotic cell death independently of p53 signaling. In addition to facilitating degradation of misfolded cytoplasmic proteins, RPN13 has also been implicated in membrane protein quality control via SGTA [56], and managing damaged mitochondria via parkin [23]. RA414 also contains a second chloroacetamide warhead that binds to cellular glutathione, which otherwise can compete for binding to RPN13 and neutralize reactive oxygen species (ROS) produced by mitochondria etc, thereby further enhancing oxidative stress and cell death. Mitophagy is a mechanism by which cells clear depolarized mitochondria mediated by parkin. Parkin binds the proteasome through the ubiquitin receptor RPN13, and RPN13 knock down/out increases parkin levels [23]. Therefore, inhibition of RPN13 is likely to promote mitophagy by stabilizing parkin. Free ubiquitin, ubiquitination and proteasomal function play critical roles in control of the DNA damage response, e.g. BRCA1 is an ubiquitin E3 ligase [57]. Since RA414 and RA413S reduce cellular levels of monomeric ubiquitin and block upstream proteasome function, the capacity to repair DNA damage caused by platinum-based chemotherapeutic agents like cisplatin is likely compromised, resulting in the observed synergistic cytotoxicity in ovarian cancer cells.

The impact of these candidate RPN13 inhibitors on the antitumor immune response is an area worthy of further study given the potential for immunogenic cell death and the role of the proteasome in antigen presentation. While the licensed 20S CS inhibitors are active against both the constitutive and immunoproteasomes [58], RPN13 is not a required component of the latter [59]. Our candidate RPN13 inhibitors enhance antigen presentation [60] as well as antitumor immune responses by counteracting myeloid suppressor cells [61]. Furthermore, even the non-canonical degradation and MHC1 processing of the ovarian cancer antigen NY-ESO-1 was only mildly impacted by knockdown of RPN13, but potently blocked by immunoproteasome inhibitors [62]. This suggests that RPN13 inhibitors may potentially trigger immunogenic tumor cell death while preserving immunoproteasome function and associated MHC1 antigen presentation better than the licensed proteasome inhibitors.

## Supporting information

S1 Fig Impact of candidate iRPN13 upon high molecular weight polyubiquitinated protein levels and NFκB signaling. (A) HeLa cells treated with compounds (0.5 μM, 3h) show high molecular weight polyubiquitinated protein accumulation measured by Western blot analysis using anti-ubiquitin monoclonal antibody. (B) same as in A, except HCT116 cells were used (C) 293T cells stably transfected with a NFκB-dependent reporter luciferase construct show dose dependent reduction in bioluminescence with drug treatment (7 h) in response to TNFα (20 ng/mL) as measured by luminometer.
(TIF)

S2 Fig Impact of RA183 on ES2 cellular bioenergetics. (A-G) Seahorse assay of ES2 cells upon treatment with 250 nM of RA183 or vehicle (DMSO) for 12 h.
(TIF)

S3 Fig RA413S and RA414 trigger apoptosis of SKOV3 ovarian cancer cells. SKOV3 cells were treated with compounds at indicated doses for 12 h and then re-suspended in 100 μL binding buffer with 5 μL of Annexin V-PE and 5 μL of 7-AAD. After a 15 min incubation at RT, the cells were analyzed by flow cytometry using a FACSCalibur and CellQuest software.
(TIF)

S4 Fig Examining synergy with chemotherapeutics approved for first and second line treatment of ovarian cancer. (A) OVCAR5 (B) PEA2 (C) PEO4 (D) SKOV3 cells treated with titrated concentrations of RA413S and cisplatin in a checker board assay as triplicates and incubated for 72 h and the cell viability was measured using MTT assay. Data were analyzed and plotted in the Synergy web finder application and Zip scores derived [40]. (E) BR5 mouse model ovarian cancer cells (*BRCA2* deficient) treated with RA413S and cisplatin (F) PEA1 cells treated with RA413S and doxorubicin (G) PEA2 cells treated with RA413S and doxorubicin (H) SKOV3 cells treated with RA414 and cisplatin.
(TIF)

S1 Table IC50 measurements for candidate iRPN13s in human cancer cell lines (nM), and the TC-1 mouse model of cervical cancer. (DOCX)

S1 Raw images Raw images of gels/blots. (PDF)

S1 File Methods for synthesis and validation of compounds. (DOCX)
