# Supplementary figures and images for: Chirality and asymmetry increase the potency of candidate ADRM1/RPN13 inhibitors
Source: PLoS One. 2021 Sep 10;16(9):e0256937. doi: 10.1371/journal.pone.0256937 (PMC8432795; doi:10.1371/journal.pone.0256937)

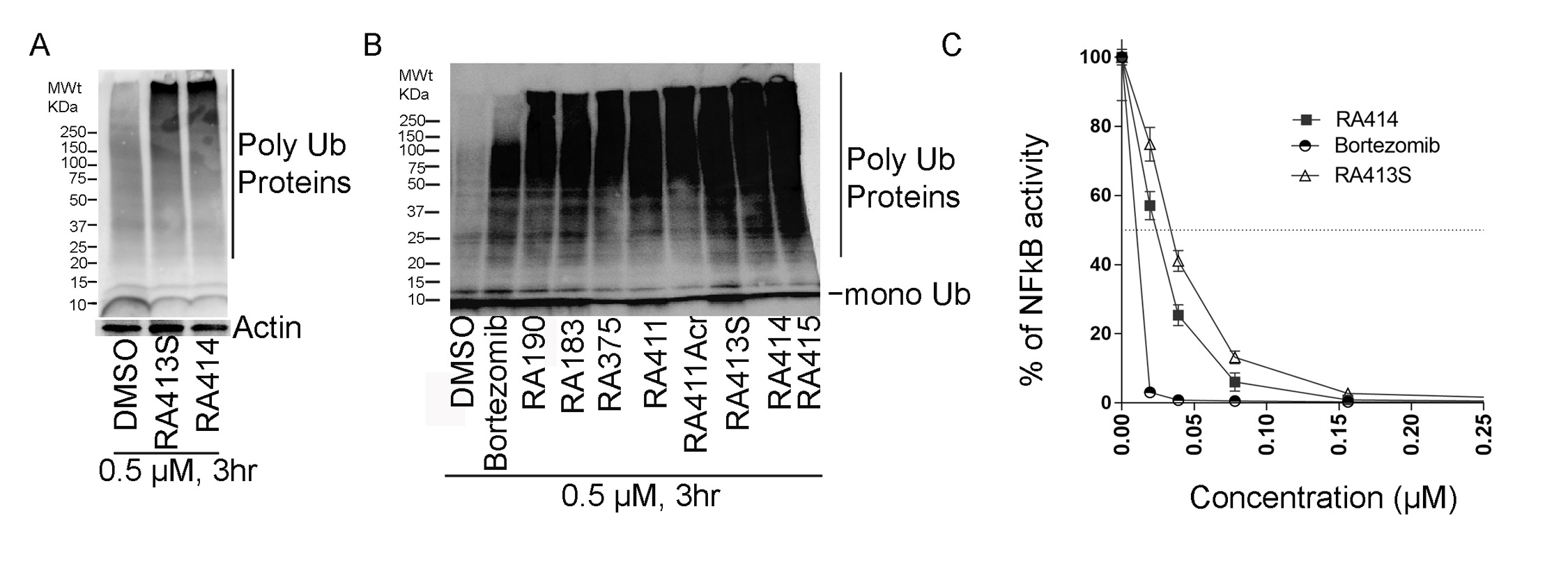

Supplement: S1 Fig — (A) HeLa cells treated with compounds (0.5 μM, 3h) show high molecular weight polyubiquitinated protein accumulation measured by Western blot analysis using anti-ubiquitin monoclonal antibody. (B) same as in A, except HCT116 cells were used (C) 293T cells stably transfected with a NFκB-dependent reporter luciferase construct show dose dependent reduction in bioluminescence with drug treatment (7 h) in response to TNFα (20 ng/mL) as measured by luminometer. (TIF) [file pone.0256937.s001.tif]

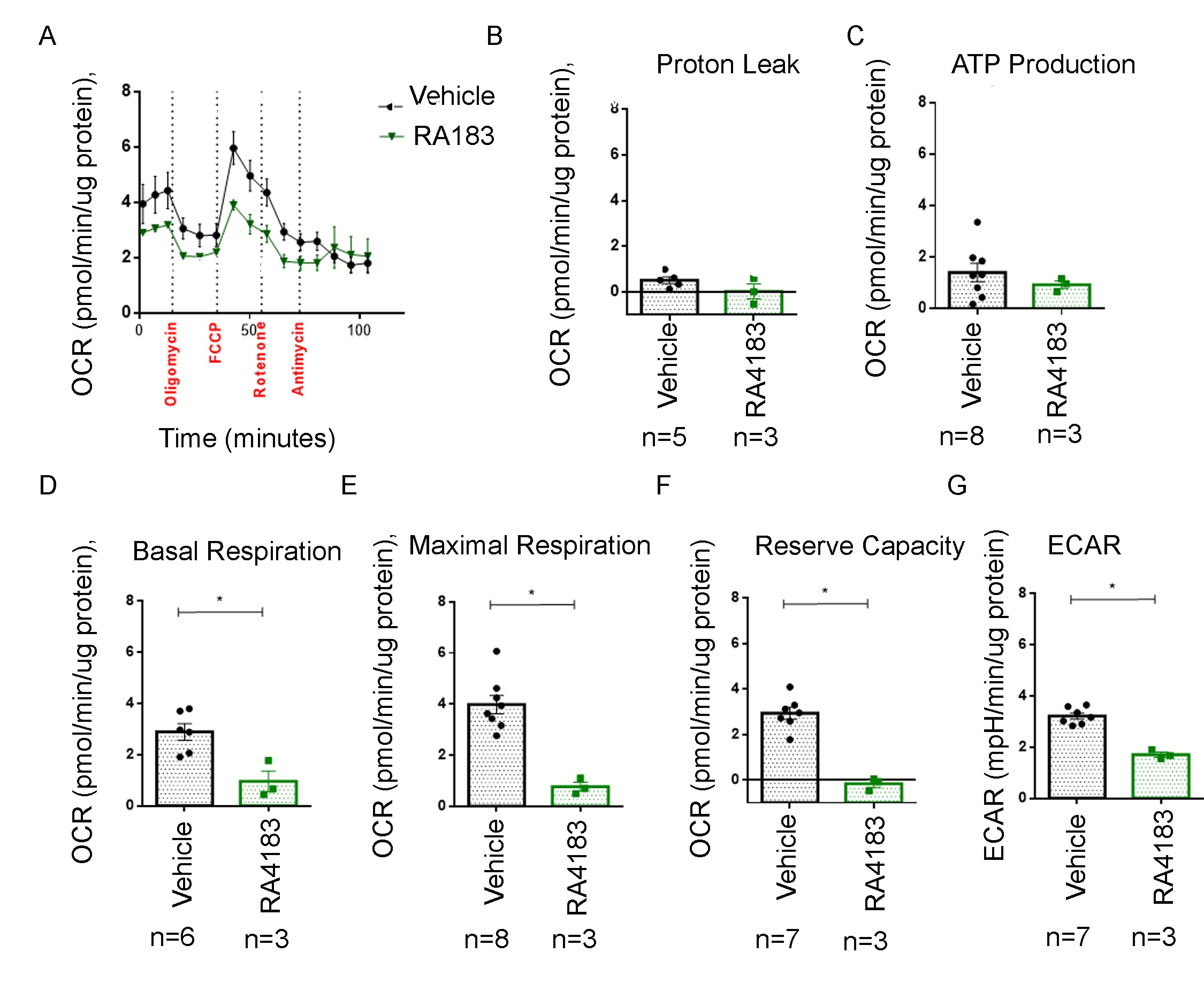

Supplement: S2 Fig — (A-G) Seahorse assay of ES2 cells upon treatment with 250 nM of RA183 or vehicle (DMSO) for 12 h. (TIF) [file pone.0256937.s002.tif]

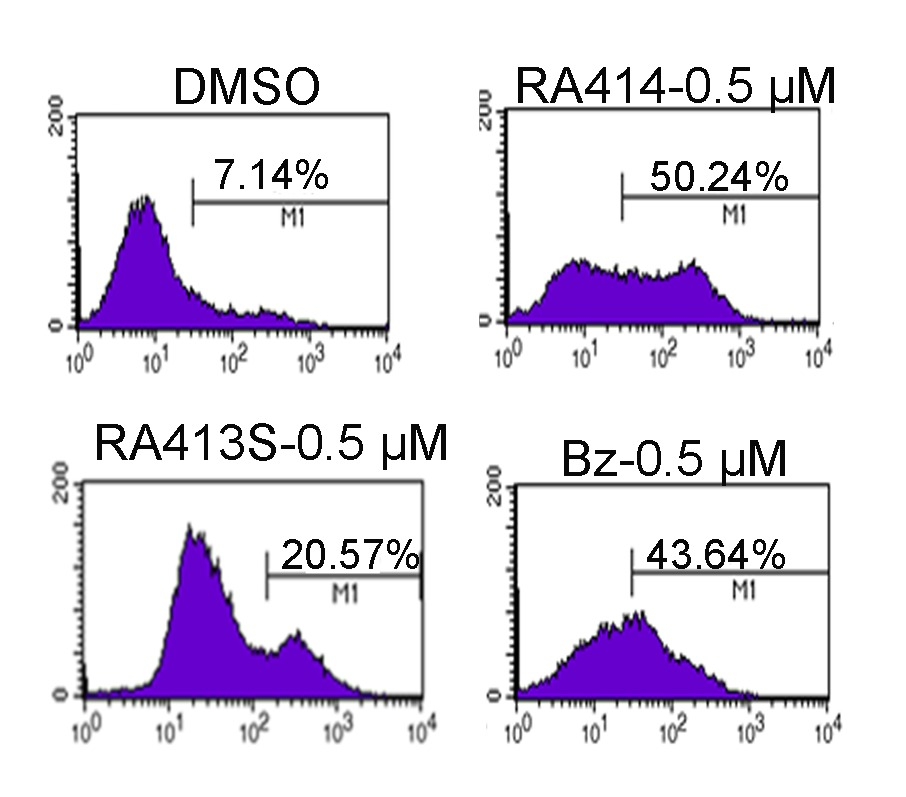

Supplement: S3 Fig — SKOV3 cells were treated with compounds at indicated doses for 12 h and then re-suspended in 100 μL binding buffer with 5 μL of Annexin V-PE and 5 μL of 7-AAD. After a 15 min incubation at RT, the cells were analyzed by flow cytometry using a FACSCalibur and CellQuest software. (TIF) [file pone.0256937.s003.tif]

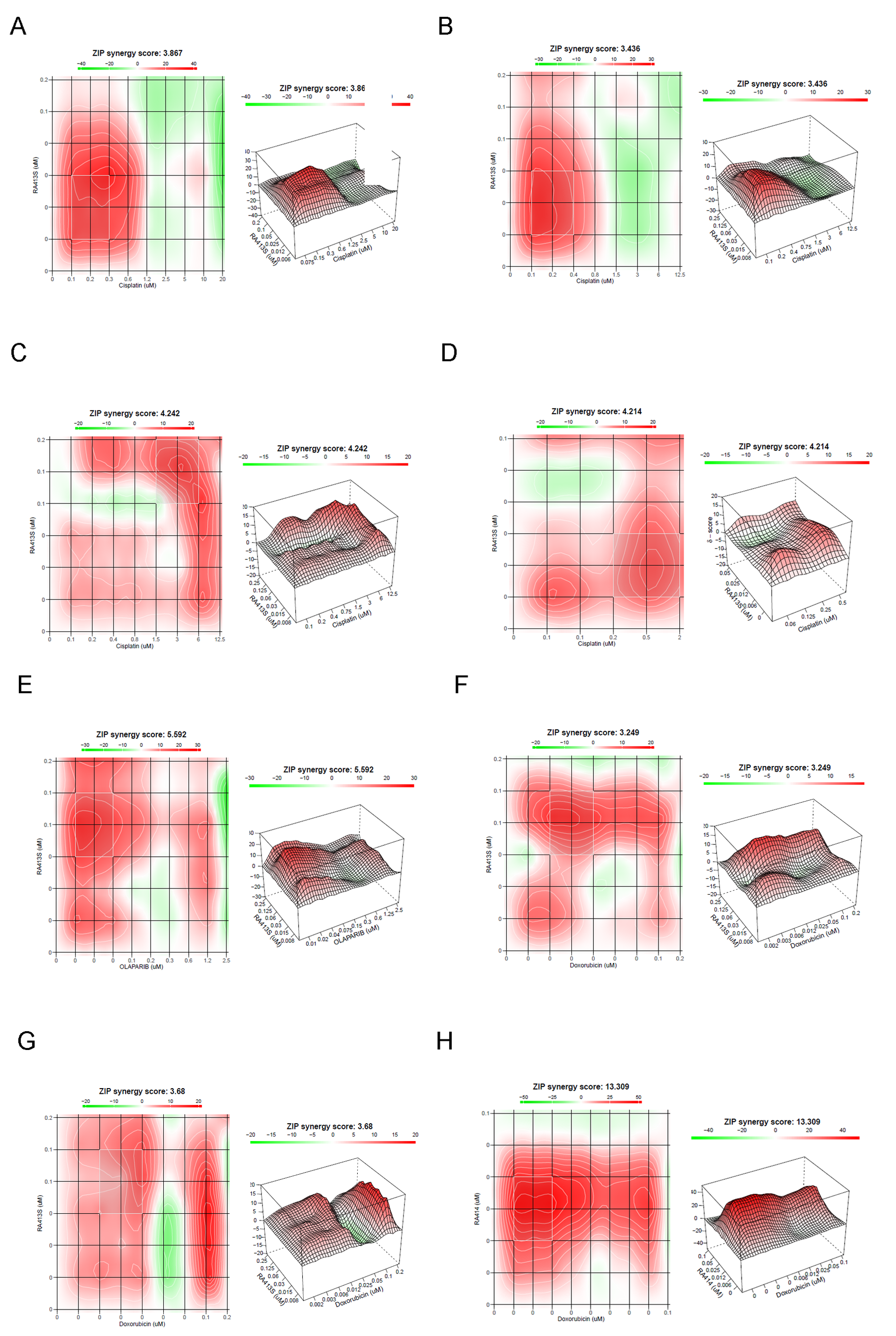

Supplement: S4 Fig — (A) OVCAR5 (B) PEA2 (C) PEO4 (D) SKOV3 cells treated with titrated concentrations of RA413S and cisplatin in a checker board assay as triplicates and incubated for 72 h and the cell viability was measured using MTT assay. Data were analyzed and plotted in the Synergy web finder application and Zip scores derived [40]. (E) BR5 mouse model ovarian cancer cells (BRCA2 deficient) treated with RA413S and cisplatin (F) PEA1 cells treated with RA413S and doxorubicin (G) PEA2 cells treated with RA413S and doxorubicin (H) SKOV3 cells treated with RA414 and cisplatin. (TIF) [file pone.0256937.s004.tif]
