# Supplementary material for: Chirality and asymmetry increase the potency of candidate ADRM1/RPN13 inhibitors
Source: PLoS One. 2021 Sep 10;16(9):e0256937. doi: 10.1371/journal.pone.0256937 (PMC8432795; doi:10.1371/journal.pone.0256937)
Supplement: S1 Table — (DOCX) [file pone.0256937.s005.docx]

**S1 Table. IC50 measurements for candidate iRPN13s in human cancer cell lines (nM), and the TC-1 mouse model of cervical cancer.**

| **Cell line name** | **Cancer**  **Type (species)** | **RA190** | **RA183** | **RA375** | **RA413S** | **RA413R** | **RA413** | **RA414** | **RA462** | **RA467** | **Cisplatin** | **Olaparib** | **Bortezomib** |
| --- | --- | --- | --- | --- | --- | --- | --- | --- | --- | --- | --- | --- | --- |
| HeLa | Cervix | 85 | 112 | 13 | 23 | 172 | 96 | 3 |  |  |  |  | 3 |
| CasKi | Cervix | 324 | 312 | 43 | 27 | 220 |  |  |  |  |  |  |  |
| SiHa | Cervix | 603 | 263 | 41 | 34 | 139 |  | 7.5 |  |  |  |  |  |
| TC1-Luc | Cervix |  |  |  | 114 | 1795 |  | 40 |  |  |  |  | 4.5 |
| DLD1 | Colon |  |  |  | 164 |  |  | <40 |  | 474 | 24790 | 23000 | 4.8 |
| DLD1 BRCA2 null | Colon |  |  |  | 55 |  |  | <40 |  | 502 | 8540 | 1869 | 2.5 |
| DLD1-Seckle | Colon |  |  |  | 55 |  |  | <40 |  |  | 964 | 14000 | 1.7 |
| HCT116 | Colon | 239 | 193 | 89 | 80 |  |  | 20 |  |  |  |  |  |
| RPE1 | Retina |  |  |  | 41 |  |  | <40 |  |  | 2086 | 23200 | 3.5 |
| RPE1-ATM null | Retina |  |  |  | 24 |  |  | <40 |  |  | 963 | 111 | 2.6 |
| SSC90 | HNSCC | 625 |  | 24 | 125 |  | 312 | <20 |  |  |  |  | 15 |
| FaDu | HNSCC | 300 | 150 |  | 78 |  | 300 | 78 |  |  |  |  |  |
| VU93 | HNSCC | 625 |  |  | 100 |  | 625 | 40 |  |  |  | 30 |  |
| HS578T | Breast | 78 | 89 | 3 | 40 |  |  | 1.5 |  |  |  |  | 13 |
| MDA-MB-468 | Breast | 300 | 250 |  | 75 |  |  | 22 |  |  |  |  |  |
| MCF7 | Breast | 750 |  |  | 250 |  |  | 75 | 750 | 750 |  |  |  |
| HCC1395 | Breast | 0.35 |  |  | 75 |  |  | <15 | 250 | 250 | 250 | 750 |  |
| SUM1315 | Breast | 1370 |  |  | 258 |  |  | 20 |  |  |  |  |  |
| SUM149 | Breast | 233 |  |  | 20 |  |  | <20 |  |  |  |  | 30 |
| HEC-1A | Endometrial | 431 | 378 |  | 207 | 268 |  | 196 |  |  | 1342 |  | 153 |
| KLE | Endometrial | 1593 | 989 |  | 1458 | 1433 |  | 1120 |  |  |  |  | 1192 |
| LNCAP | Prostate | 242 | 186 | 60 |  |  |  | 24 |  |  |  |  |  |
| DU145 | Prostate |  |  |  |  |  |  | 17 |  |  |  |  |  |
| PC3 | Prostate | 162 | 133 | 27 |  |  |  | 12 |  |  |  |  |  |
