# Supplementary material for: Chirality and asymmetry increase the potency of candidate ADRM1/RPN13 inhibitors
Source: PLoS One. 2021 Sep 10;16(9):e0256937. doi: 10.1371/journal.pone.0256937 (PMC8432795; doi:10.1371/journal.pone.0256937)
Supplement: S1 Raw images — (PDF) [file pone.0256937.s006.pdf]

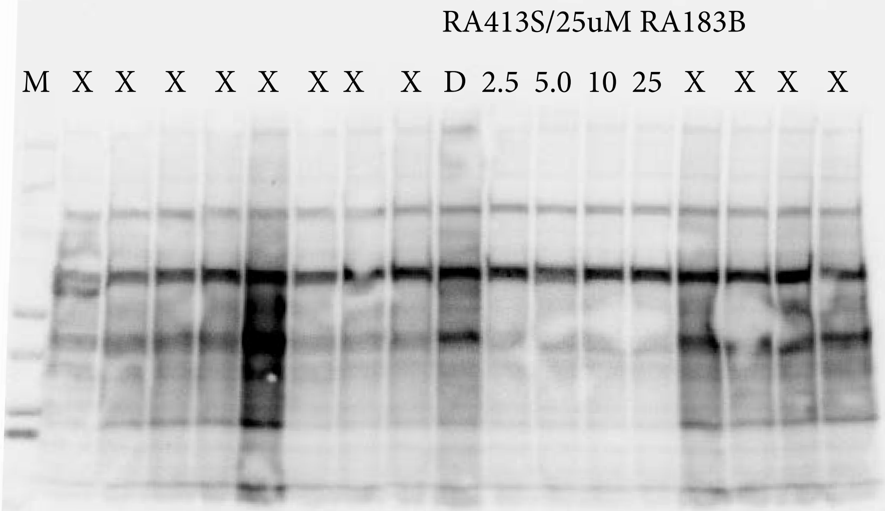

Figure 3A taken with Biorad Chemidoc Touch

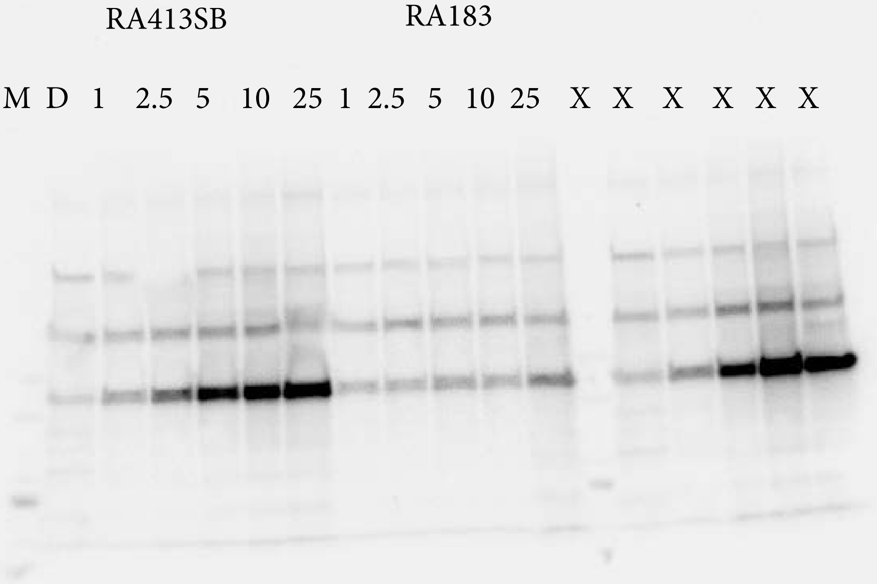

Figure 3B taken with Biorad Chemidoc Touch

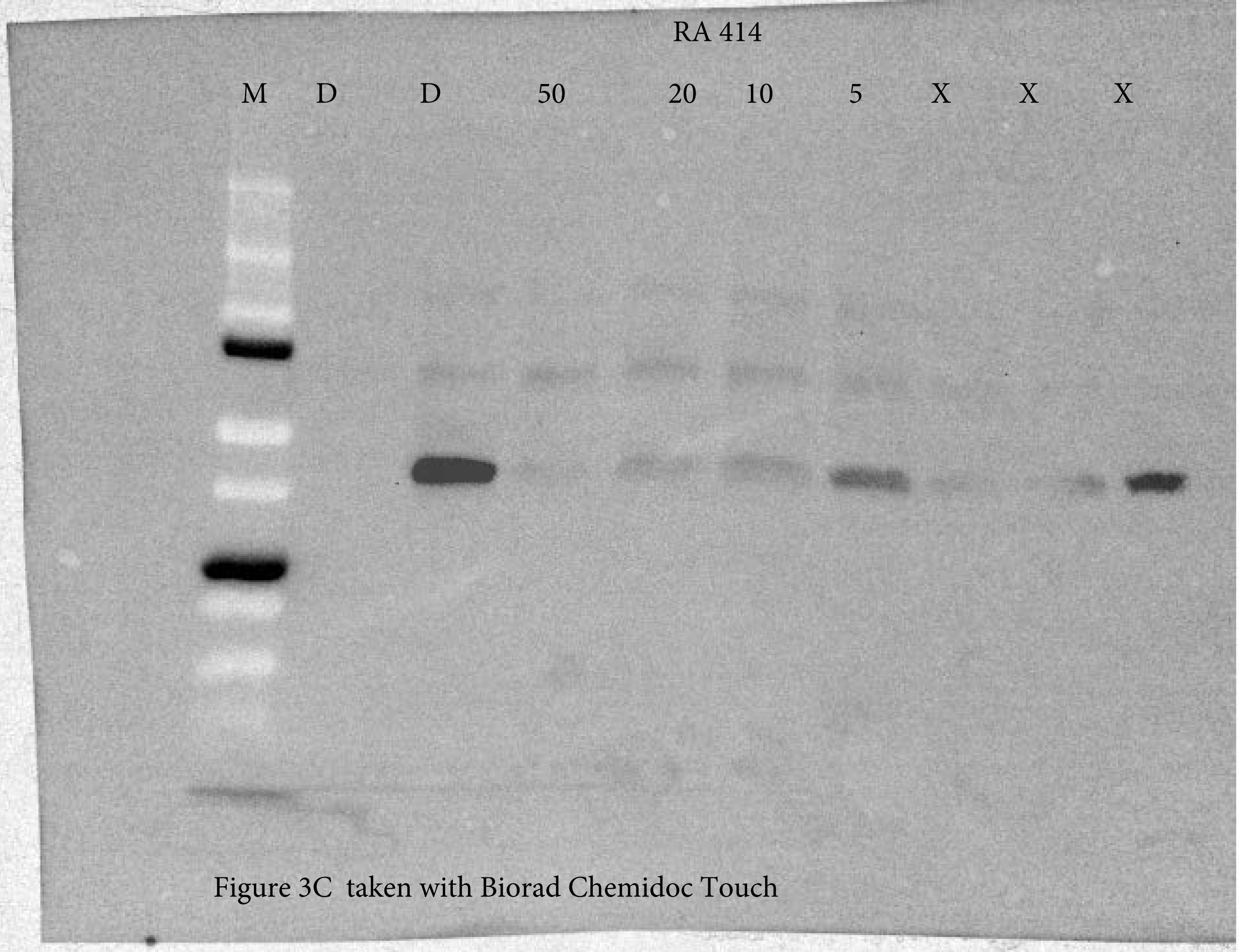

Figure 3C taken with Biorad Chemidoc Touch

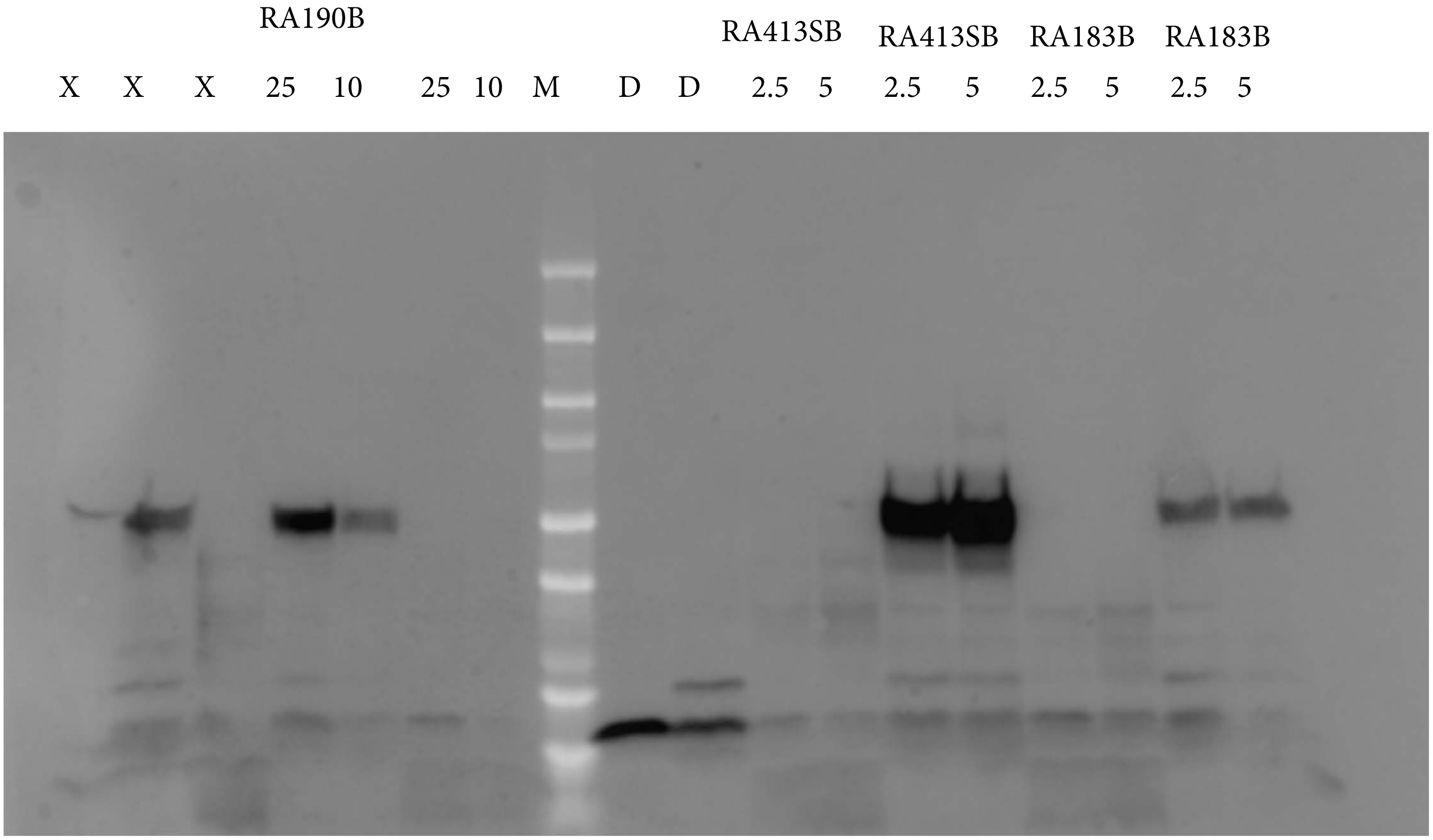

Figure 3D taken with Biorad Chemidoc Touch

Figure 4A taken with Biorad Chemidoc Touch

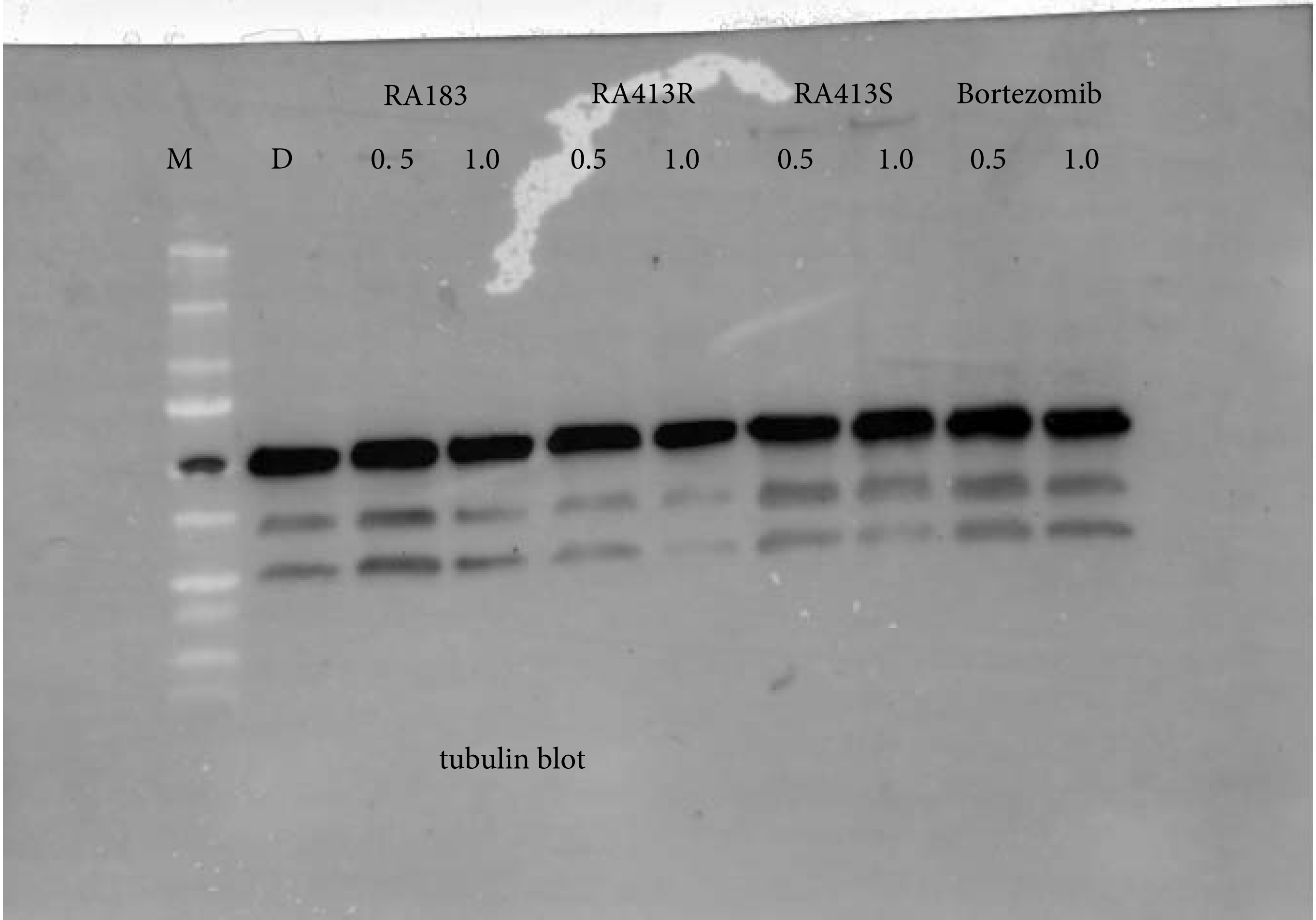

Figure 4A taken with Biorad Chemidoc Touch

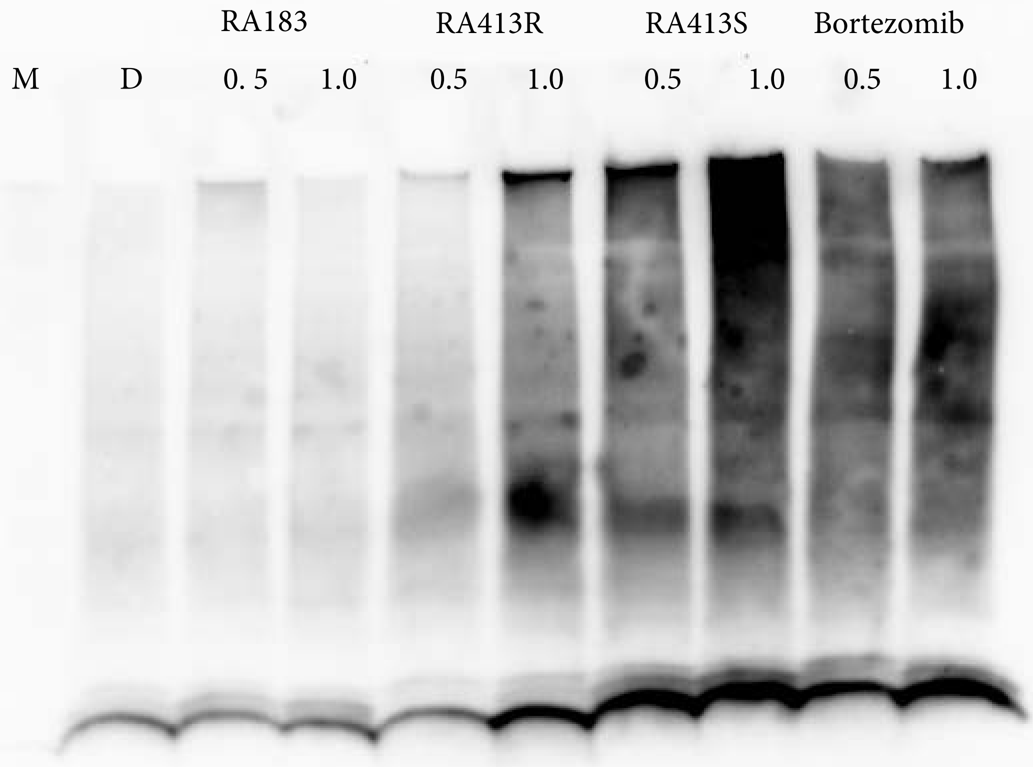

Ubiquitin blot

Figure 7 taken with Biorad Chemidoc Touch

Actin blot

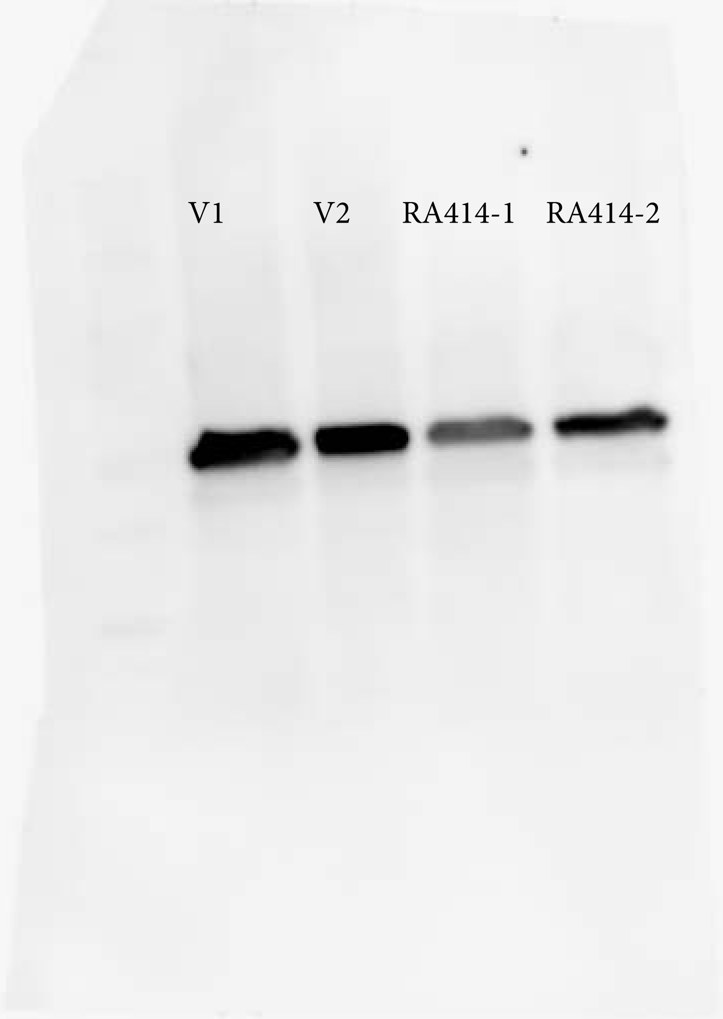

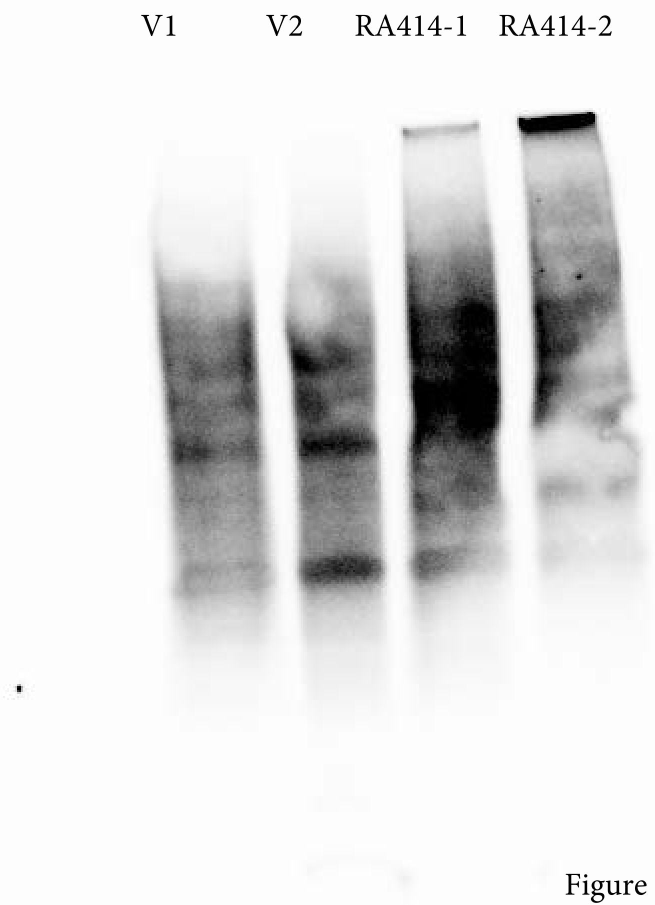

Figure 7 taken with Biorad Chemidoc Touch

Ubiquitin blot

Ubiquitin Blot

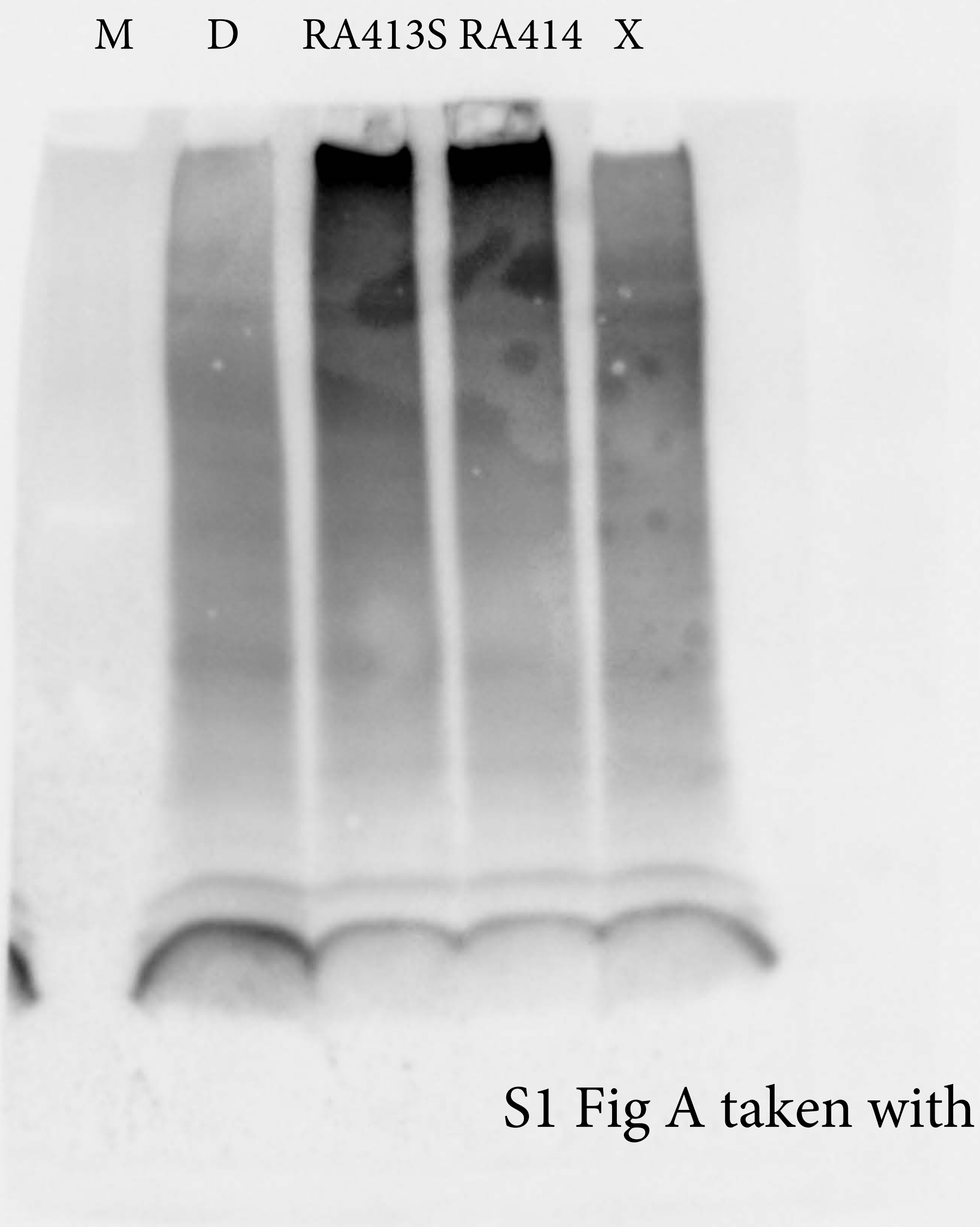

S1 Fig A taken with Biorad Chemidoc Touch

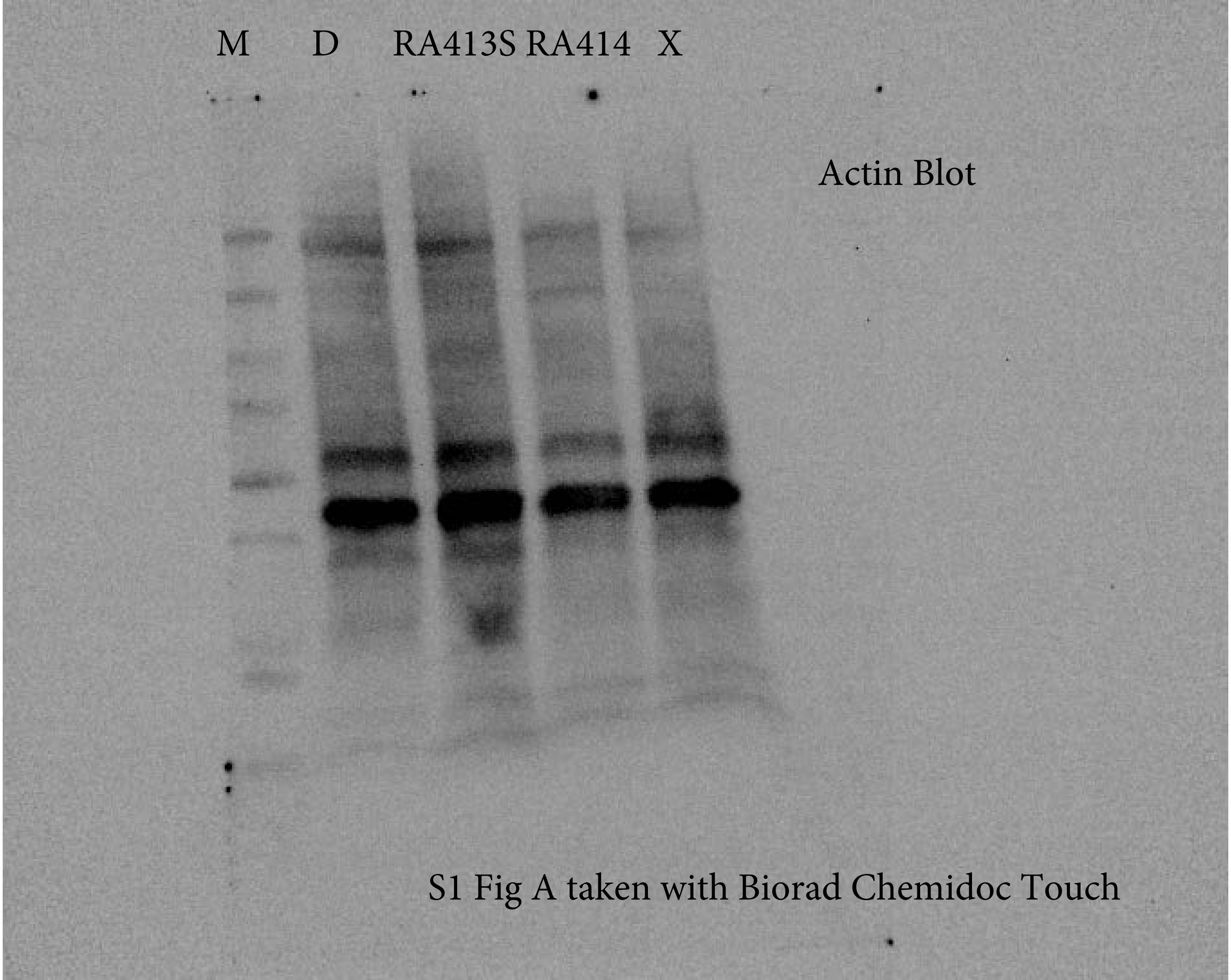

S1 Fig A taken with Biorad Chemidoc Touch

S1 Fig A,Biorad Chemidoc Touch

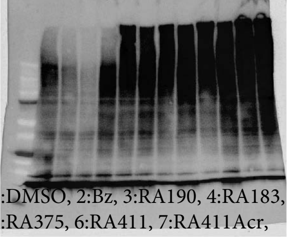

1:DMSO, 2:Bz, 3:RA190, 4:RA183, 5:RA375, 6:RA411, 7:RA411Acr, 8:RA413S, 9:RA414, 10:RA415
