# Supplementary material for: Chirality and asymmetry increase the potency of candidate ADRM1/RPN13 inhibitors
Source: PLoS One. 2021 Sep 10;16(9):e0256937. doi: 10.1371/journal.pone.0256937 (PMC8432795; doi:10.1371/journal.pone.0256937)
Supplement: S1 File — (DOCX) [file pone.0256937.s007.docx]

**S1 File. Methods for synthesis and validation of compounds.**

**General:** 1H and 13C NMR spectra were recorded as a CDCl_3_ or CD_3_OD or DMSO-d6 solution on a Bruker ADVANCE 400 FT NMR operating at 400 MHz. Chemical shifts are reported in parts per million. J values were measured in Hz. All commercially available materials and solvents were purchased from Sigma Aldrich or Fisher scientific.

**Synthesis of RA413S:** (a) 4-NO_2_-CHO, AcOH, dry Hcl gas, overnight (b) DMF, Diisopropy Ethyl amine, Boc-Phe-OH, HBTU,HOBt, overnight (c) 4M HCl in dioxane, 30 min

**Synthesis of RA414:** (a) 4-NO_2_-CHO, AcOH, dry HCl gas, overnight (b) DMF, Diisopropy Ethyl amine, Boc-Phe-OH, HBTU,HOBt, overnight (c) 4M HCl in dioxane, 30 min (d) DCM, Diisopropyl ethylamine, chloroacetyl chloride, 0^0^C- rt, 30 min

***Preparation of compound RA411S:*** *(S)-2-methyl-3,5-bis((E)-4-nitrobenzylidene)piperidin-4-one acetate*

A stirred solution of compound 1 (1 mmol) in acetic acid (10 mL) was added 4-nitro-benzaldehyde (2 mmol) and then dry HCl gas was passed into the solution for 30 min continuously while stirring. The resulting yellow colored solution was left over night without stirring and the resulting precipitate was filtered and washed with cold ethanol and filtered and dried to afford compound RA411S. ^1^H NMR (DMSO-d6) δ1.61 (s, 3H), 4.7 (dd, 2H, J = 8.0 Hz), 4.99-5.08 (m, 1H), 7.88-8.03 (m, 10H); m/z ES+1: 380

***Preparation of compound RA412S:*** *tert-butyl ((S)-1-((S)-2-methyl-3,5-bis((E)-4-nitrobenzylidene)-4-oxopiperidin-1-yl)-1-oxo-3-phenylpropan-2-yl)carbamate*

A stirred solution of Boc-Phe-OH (1 mmol) in DMF (10 mL) was added HBTU (1.1 mmol), HOBT (1.1 mmol) and diisopropylethylamine (3 mmol) at 0^0^C. To this reaction mixture RA411S (1 mmol) in DMF (1 mL) was added and reaction was stirred at room temperature for overnight. After completion of the reaction, as indicated by TLC, the solvent was removed under reduced pressure and the resulting crude was partitioned between ethyl acetate (2X50 mL) and water. Ethyl acetate layer was washed with brine and dried over sodium sulfate. Removal of ethyl acetate under reduced pressure and column chromatography purification using Ethyl acetate and hexanes as eluents (40:60) afforded yellow powder.

***Preparation of compound RA413S:*** *(S)-1-(L-phenylalanyl)-2-methyl-3,5-bis((E)-4-nitrobenzylidene)piperidin-4-one hydrochloride*

A solution of RA412S (1 mmol) in 4M HCl in dioxane (10 mL) was stirred for 30 min at room temperature. After completion of the reaction, as indicated by TLC, dioxane was removed under vacuum and the resulting slurry was added to diethyl ether to precipitate yellow solid. Precipitates were filtered and washed three times with diethylether and dried overnight under vacuum to afford RA413S as HCl salt. ^1^H NMR (400MHz, CD_3_OD) δ1.4 (s, 3H, CH3), 2.6-3.1 (m, 2H, CH2), 4.32-4.81 (m, 4H, -CH, -NCH, Ph-CH2), 6.81 (s, 1H, -C=CH), 7.01 (s, 1H,-C=CH), 7.1-7.92 (m, 8H), 8.23-8.52 (m, 5H); 13C NMR (400MHz, CD_3_OD); δ 19.2, 39.1, 42.2, 44.6, 52.4, 126.1, 129.8, 143.3, 146.9, 151.2, 170.3, 189.7; m/z ES+1: 527. Based on the NMR and MS analyses, the purity of the compound was >95%.

***Preparation of compound RA414:*** *2-chloro-N-((S)-1-((S)-2-methyl-3,5-bis((E)-4-nitrobenzylidene)-4-oxopiperidin-1-yl)-1-oxo-3-phenylpropan-2-yl)acetamide*

Racemic RA413 was synthesized as for RA413S. To a solution of racemic RA413 (1mmol) in dichloromethane (DCM) (20 mL) was added diisopropylethyalamine (3 mmol) and chloroacetylachloride (1mmol) at 0 ^°^C and the reaction mixture stirred for 30 min. After completion of the reaction as indicated by TLC, DCM was partitioned with water, separated, washed with brine and dried over sodium sulfate. DCM was removed under reduced pressure and the crude compound was purified by silica gel column chromatography using DCM:Acetone (96:4) as solvents to afford racemic RA414 as a yellow powder. ^1^H NMR (400MHz, CDCl_3_) δ1.6 (s, 3H,-CH3), 3.1 (dd, 2H, -CH2, J=8Hz), 3.92-4.08 (m, 2H, Ph-CH2), 4.14 (s, 2H, -CH2-Cl) 4.45 (dd, 1H, -CH, J=16Hz), 4.98-5.09 (s, 1H, N-CH), 7.01-7.9 (m, 10H, aromatic and –C=CH), 8.3-8.45 (m, 5H, phenylalanine aromatic ring). m/s ES: 603.3. Based on the NMR and MS analyses, the purity of the racemic RA414 is >95%. The NMR analysis showed a mixture of diasteriomers.
